# Synthesis of anisotropic gold nanoparticles in binary surfactant mixtures: a review on mechanisms of particle formation

**DOI:** 10.1039/d4ra06358a

**Published:** 2025-02-10

**Authors:** Katharina Ruth Zürbes, Ethayaraja Mani, Sulalit Bandyopadhyay

**Affiliations:** a Particle Engineering Centre, Department of Chemical Engineering, Norwegian University of Science and Technology N-7491 Trondheim Norway sulalit.bandyopadhyay@ntnu.no; b Polymer Engineering and Colloid Science Lab, Department of Chemical Engineering, Indian Institute of Technology Madras Chennai 600036 India

## Abstract

Gold nanoparticles are promising candidates for a wide spectrum of biomedical applications ranging from diagnostics and sensors to therapeutics. Their plasmonic properties are dependent on their size and shape among other factors, which can be controlled by understanding various growth mechanisms. Since the breakthrough of the seed-mediated growth approach reported in 2001, the scientific community has actively engaged in the synthesis of tailored anisotropic gold nanoparticles. Surfactants are known for their shape-controlling abilities and since Nikoobakht and El-Sayed in 2003 used a binary surfactant mixture, this method has been adopted as a common synthesis strategy. A wide range of particle shapes have been produced in binary surfactant mixtures using different synthesis approaches, and different working mechanisms have been proposed. This calls for a thorough and critical evaluation of the synthetic methods with an aim to bridge the link between the use of binary surfactants and the control of morphology of anisotropic gold nanoparticles. This review gives a systematic overview of experimental procedures using binary surfactant mixtures to produce gold nanoparticles with tuned properties. The resulting shapes include gold nanorods, bipyramids, tetrahexahedra, and other anisotropic structures. Different mechanisms proposed based on experimental, simulation and modelling analyses are discussed based on the type of binary surfactant systems. Current challenges that need to be addressed and future prospects that may open up new avenues of applications for anisotropic gold nanoparticles are also discussed.

## Abstract 2

Overview of gold nanoparticle synthesis using binary surfactants: key mechanisms and future directions.

## Graphical abstract

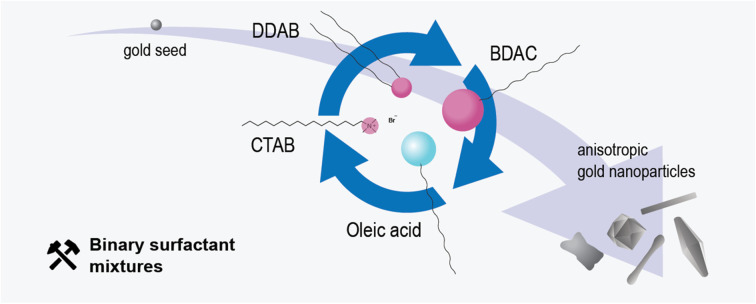

## 1 Introduction

The introduction of shape anisotropy in gold (Au) nanoparticles (NPs) has a dramatic effect on their optical properties, specifically on their localized surface plasmon resonance (LSPR). Anisotropy splits up the single LSPR band, generally observed for spherical Au NPs, into at least two distinct peaks, representative of the transverse and longitudinal axes. Fine controlling the aspect ratio (AR; defined as the ratio of length to width) of Au NPs has become important, as this enables a wide range of optical properties, ranging from optical imaging to photothermal applications, to be explored by changing the AR.

In designing such NPs with different shapes and sizes, the seed-mediated growth approach has been demonstrated as a powerful synthetic route.^1,2^ The underlying principle for this approach comprises two main steps-in the first step, tiny Au seed particles are generated at high values of supersaturation by using a strong reducing agent and in the second step, a milder reducing agent is used to favor directed growth of these seed particles in the presence of surfactants,^3^ organic additives or binary surfactant mixtures^4–10^ while maintaining low levels of supersaturation. Separating nucleation from growth enables better control of reaction parameters to obtain particles with well-defined morphologies.

Through this approach, the growth of Au NPs of different shapes has been investigated in the presence of the lone surfactant cetyltrimethylammonium bromide (CTAB) with or without growth directing species such as silver.^1^ Although several works provide a mechanistic understanding of anisotropic Au NPs synthesis *via* this method, it is challenging to synthesize anisotropic Au NPs with high monodispersity and size/shape tunability is restricted. To overcome these limitations, a modified seed-mediated growth method was proposed based on the use of binary surfactants or organic additives in the growth solution.^4^ Since then, this research field has seen an exponential increase in the use of binary surfactants to control growth and in turn, synthesize novel morphologies with enhanced optical properties.

Since the surfactant plays a central role in the shape evolution of the nanoparticles, use of a binary surfactant mixture broadens and eases the synthesis of colloidal gold nanoparticles, while simultaneously complicating the mechanistic understanding of size and morphology control, among others. This calls for a comprehensive and systematic review of the so far published works focusing on synthesis employing binary surfactant mixtures leading to anisotropic colloidal gold nanoparticles.

The aim of this review is, therefore, to promote the understanding of the experimental progress by undertaking a thorough and critical summary of already published works in this field as well as systematize the proposed underlying mechanisms by comparing and contrasting with mechanisms in a single surfactant system. Efforts have also been concerted here to understand the growth mechanisms from modelling and simulation studies reported in the literature. Further, the review will throw light on how these different morphologies may be rationally designed to tailor them for desired biomedical applications in the future. This review gives a short introduction to surfactants and micelles followed by a look into the nanoparticle formation principles, the synthesis approaches for gold nanoparticles and is providing a comprehensive overview on the synthesis conditions and resulting properties of anisotropic gold nanoparticles, ordered by the binary surfactant mixture system. It is further mapping the different proposed growth mechanisms in these systems from a simulation and an experimental perspective and proposes future steps to exploit binary surfactants.

### 1.1 Surfactants and micelle formation

Surfactants, such as cetyltrimethylammonium bromide (CTAB) are known to adsorb onto interfaces due to their amphipathic nature,^11,12^ resulting in reduction of interfacial free energy. CTAB has played a pivotal role in the synthesis of anisotropic gold nanoparticles. CTAB and other surfactants self-organize as association colloids called micelles at and above CMC (critical micelle concentration) driven primarily by entropy. The process of micellization is also governed by temperature, especially for ionic surfactants, whose solubility is strongly temperature dependent. Although spherical micelles are most commonly studied in the literature, association colloids can take other shapes which is determined by the critical packing parameter (CPP)^13^ as defined below, where *v*_t_ represents the volume of the hydrophobic chain, *a*_h_ is the effective head group area and *l*_c,t_ is the length of the hydrophobic chain.
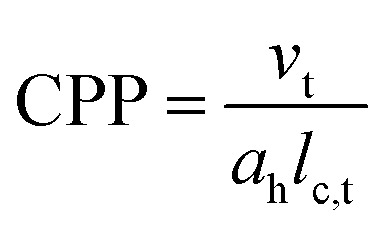


**Fig. 1:**
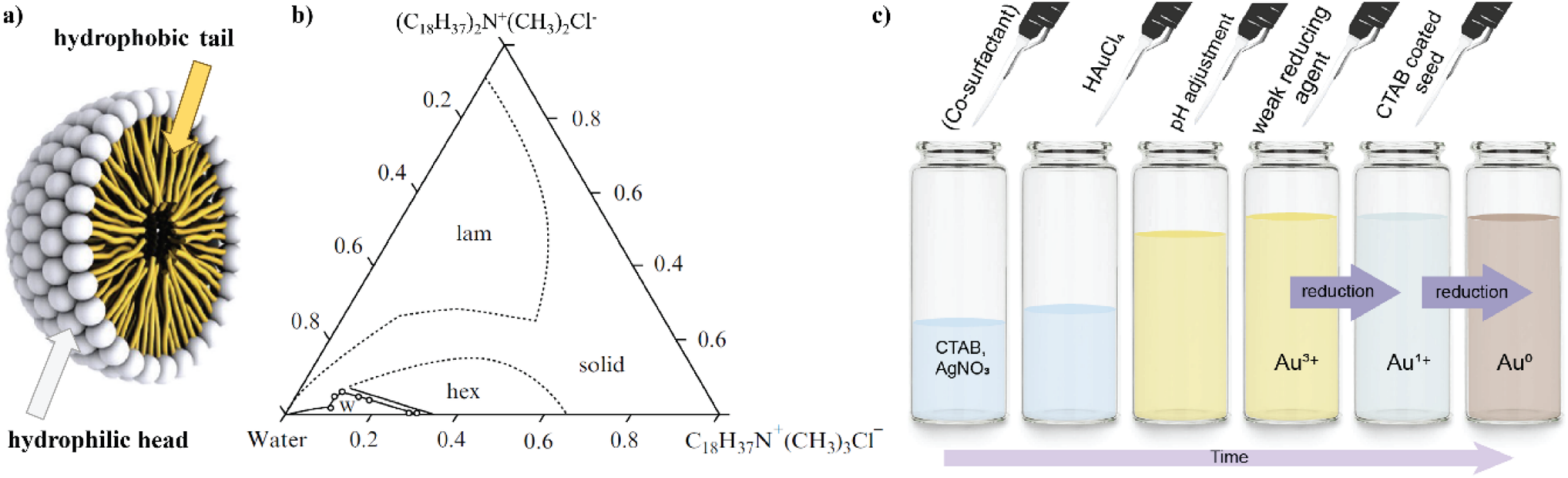
(a) Bisection of a micelle showing the steric organization. It is characterized by tail-to-tail and head-to-head ordering; [reproduced and adapted from ref. 14, Copyright 2012, Bitounis *et al.*, licensed under CC-Y 3.0 (Creative Commons — Attribution 3.0 Unported — CC BY 3.0)]; (b) ternary phase diagram of two cationic surfactants: the double-chain bilayer forming surfactant (C_18_H_37_)_2_N^+^(CH_3_)_2_Cl^−^ and the single-chain micelle-forming surfactant C_18_H_37_N^+^(CH_3_)_3_Cl^−^. The phase behavior can be understood with the help of an average critical packing parameter (CPP) at the different surfactant ratios; [adapted with permission from ref. 15 and 16 Copyright 1978, American Chemical Society]. (c) Schematic preparation of a growth solution for silver-assisted seed-mediated growth towards colloidal anisotropic gold nanoparticles.

For a spherical micelle, the CPP must be below 1/3 (see bisection of a spherical micelle in Fig. 1a), while for cylindrical micelles, which can form hexagonal arrangements, CPP must lie between 1/3 and 1/2. At CPP close to 1, the aggregates form planar bilayers and inverted micelles are formed when CPP exceeds 1.

CPP also suffices the prediction of changes in the aggregate structures for example upon changing the pH, electrolyte concentration, the charge of the headgroup or the length of the hydrophobic tail. Additionally, several of these association structures may be formed by the introduction of a third phase, like an oil into a water-surfactant mixture. Some of these interesting properties for the most commonly used surfactants in gold nanoparticle synthesis are listed in the Table 1 below.

**Table 1:** Characteristic properties of surfactants which are used for gold nanoparticle synthesis using binary surfactant mixtures; notation for the headgroup charge: + (cationic); − (anionic); ° (not charged)

Short name	Chemical name	Head group size	CPP	Head group charge	CMC in water
BDAC	Benzyldimethylhexadecylammonium chloride	—	—	+	0.6 mM (ref. 17)
CTAB	Cetyltrimethylammonium bromide	0.69 nm^2^ per molecule [RNMe_3_]^+^ headgroup: 0.521 nm^2^ (ref. 2)	0.38, 25 °C; 0.36, 35 °C (ref. 18)	+	1.0^a^ mM (ref. 19); 0.97 mM, 25 °C; 1.14, 35 °C (ref. 18)
CTAC	Cetyltrimethylammonium chloride	0.627 nm^2^ per molecule (ref. 20)	0.33 (ref. 20)	+	1.6 mM (ref. 20)
DDAB	Didodecyldimethylammonium bromide	75 Å^2^ (ref. 21)	0.56 (ref. 21)	+	CVC = 0.165 mM (ref. 21); CVC = 7.29 ± 0.02 103 mol dm^−3^, 35 °C (ref. 22)
F-108	Pluronic	—	—	°	2.2 × 10^−5^ M, 37 °C (ref. 23)
L-64	Pluronic	—	—	°	4.8 × 10^−4^ M, 37 °C (ref. 23)
NaOL	Sodium oleate	30–50 Å^2^ per molecule (ref. 24)	—	−	4.7 × 10^−2^ mM at pH 7 (ref. 24)
SDSn	Sodium docecylsulfonate	—	SDSn – CTAB phase diagram (ref. 25)	−	9.87 mmol kg^−1^ (ref. 26); 10.8 mmol kg^−1^, 25 °C, 10.6 mmol kg^−1^, 35 °C

a Measured with tensiometry method.

The shape tunability of the micelles may also be achieved by the addition of a second surfactant. Depending on the charges of the two surfactants as well as other parameters, their interaction is influenced. Especially mixtures from two surfactants with the same charge can often be described in terms of their mixed micellar properties as a linear combination of the single surfactants. The different aggregation phases can be conveniently mapped in a ternary phase diagram, like displayed in Fig. 1b. For oppositely charged surfactants however, the resulting mixtures are not as straightforward to understand. An often observed phenomenon is however that head group charge screening of the oppositely charged surfactant molecules leads to higher packing density in these mixed micelles.^12,15,16^ Further, there is a class of molecules called co-surfactants. These molecules can for example be short-chained amines, alcohols^27^ or organic acids.^10^ The term is used here for molecules which influence the properties of the primary surfactant in the mixed micelle.

### 1.2 Basics of crystal nucleation and growth

The development of metallic nanoparticles in a bottom-up approach can be described through the principles of classical crystal nucleation and growth.^28,29^ In homogeneous nucleation, the nuclei form from a pure solution of the parent phase driven by the reduction of the total free energy comprising surface free energy and the bulk free energy. During the nanoparticle formation, the sum of these two energies first increases, until the critical radius of the nucleus is reached. After this point it is thermodynamically advantageous if the nucleus continues growing.^28–30^ In contrast, heterogeneous nucleation has an energetic advantage as the nuclei are forming on structural inhomogeneities like impurities and grain boundaries, which lowers the energy barrier for nucleus formation.^28,29^ Further, the nucleation can also be understood from other perspectives where for example pre-nucleation clusters (PNC) are formed in contrast to the monomer attachment suggested from the classical nucleation theory.^28,31^

After the formation of the nucleus, crystal growth proceeds, which results in size enlargement as well as development of distinct morphology or external shape or habitus. The classical growth theory is based on the continuous addition of monomer building units onto the nucleus or growing (nano)particle following the lattice structure. The resulting morphology, following this theory, is dictated by the thermodynamic driving force of energy minimization. Growth proceeds following two steps: diffusion of growth units towards the crystal surface and incorporation of the same into the growing particle by a surface reaction. Both steps are highly dependent on the solution supersaturation of the growth units and can be described with the LaMer diagram.^29,32–34^ Again, in addition to this classical growth pathway, other mechanisms involving formation of mesocrystals or oriented aggregation of primary nanoparticles and subsequent fusion to a single crystal have been proposed.^31,32,35,36^ A special mode for crystal growth is the seed-mediated growth, where nucleation is spatially and temporally separated from the subsequent growth. This growth method has enabled the synthesis of anisotropic gold nanorods with a high shape yield, which is commonly used today.^1,30^

## 2 Synthesis of anisotropic gold nanoparticles

### 2.1 Single surfactant

As shown by Lohse and Murphy^1^ the development of the seeded-growth method for the synthesis of colloidal gold nanorods published in 2001 (ref. 37) marks the starting point to a soon to be flourishing research field. Especially, the introduction of silver in the growth solution, published in 2003,^4^ enabled a one-step growth method, which made the gold nanorod synthesis easy and ready to adapt and improve. A classical growth solution preparation is displayed in Fig. 1c, which shows the addition of the pre-prepared gold seeds as the last step before nanoparticle growth.

On the other hand, a so-called seedless or one-pot synthesis was published in 2005,^38^ where the seed is generated *in situ* in the growth solution by the addition of a strong reducing agent like NaBH_4_. This approach is facilitated in one reaction vessel and allows for gram-scale reaction yield. However, the monodispersity is low compared to the seeded growth method and in general one is constrained to rather small nanoparticles (diameter less than 10 nm).^39^ Lastly, the nanoparticles achieved from the above-mentioned methods can also be used as ‘new seeds’ in overgrowth or regrowth reactions to alter their morphology, size and monodispersity.

General goals for the synthesis of colloidal anisotropic gold nanoparticles are a monodisperse product, with low impurities from other shapes (so a high shape yield). Anisotropic gold nanoparticles further entail interesting optical properties. These can be harnessed most effectively when the resulting nanoparticles show a narrow LSPR peak, representing monodisperse particles that can for example give a higher photon efficiency in photothermal therapy or enable uniform biosensing amongst others. A superior way to achieve these goals is by using binary surfactant mixtures in the growth solution. Using two different surfactants enables enhanced shape tunability and subsequent process intensification resulting in better growth control.^40^

### 2.2 Binary surfactants

The ratio of different reactants in the synthesis of gold NPs is a powerful tool to direct the shape of the resulting particles^10^ and in this regard, a second surfactant in addition to the commonly used CTAB forms the basis of growth using binary surfactant mixtures.^4,37,41,42^ Synthesis routes with different binary surfactant mixtures and the key properties of the resulting gold nanoparticles are presented ordered by their surfactant ratios (Table 2). This review does not consider aromatic additives^43,44^ or PVP^45^ as (co-) surfactants nor the use of gemini surfactants.^46^

**Table 2:** Overview of shapes resulting from a growth solution containing a binary surfactant mixture and indication of presence of Ag(i) in the growth solution with their shape yield, sizes, aspect ratios and longitudinal localized surface plasmon resonance (LSPR); abbreviations for the different morphologies: NR: nanorod; BP: bipyramid; CCNCB: concave nanocuboids; THH: tetrahexahedra; NM: nanomakura; CVNCB: convex nanocuboids; fwhm: full width at half maximum; aspect ratio describes the proportion between the long and the short axis of a gold nanoparticle

Morphology	Surfactant ratio; Ag(i)^a^	Width (*W*) and length (*L*) size and aspect ratio (AR)^b^	LSPR wavelength	Comments with focus on seed type and shape yield	Ref.
**1. CTAB : BDAC**					
1.1 NR	1 : 1.25; +	*W*: 11–22 nm	700–1020 nm	CTAB seeds; higher aspect ratio when early addition of the co-surfactant BDAC	7
		*L*: 55–73 nm			
		AR: 2.2–6			
1.2 NR	1 : 1.4–7; +	*W*: 6–12 nm	700–1300 nm	CTAB seeds; first paper using binary surfactant mixtures for seed-mediated growth of gold nanoparticles; surfactant ratio of 2.7 suppresses the formation of spheres; take 200 particle sizes into account from TEM images	47
		AR: 4.6–10			
1.3 NR	1 : 2.5; +	*L*: 42 nm	**—**	CTAB seeds; the authors do not state the individual concentrations of the surfactants, only refer to the surfactant ratio	48
		AR: 5.6			
1.4 NR	1 : 0.7–5; +	*W*: 10–17 nm	664–967 nm	CTAB seeds; highest aspect ratio with CTAB : BDAC ratio 1 : 1	49
		*L*: 38–72 nm			
		AR: 2.5–6			

**2. CTAB : CTAC**					
2.1 Regrown BP	90 : 1–1:9000; +	*W*: 27–31 nm	700–1100 nm	Bipyramids (grown from citrate seeds) were used for regrowth; tip regrowth in surfactant ratio CTAB : CTAC 1 : 90–9000; polydispersity: 3.7–5.3%	8
		*L*: 84–137 nm			
		AR: 2.8–4.8			
2.2 NR	1 : 1.25; +	*W*: 17–25 nm	*ca.* 780 nm	CTAB seeds; higher shape yield when the co-surfactant CTAC was added later in the reaction	7
		*L*: 35–55 nm			
		AR: *ca.* 1.9–2.1			
2.3 Spinous-like	1 : 0.25–4; −	300–400 nm – (no longer dimension)	—	No details were given for the formation of the CTAC coated seeds	50
2.4 “Au nanoarchitectures” – with nanotips	0.0125–7 : 1; −		*ca.* 900 nm	Morphologies described as meatball, star-like and spherical like	51
				30 nm gold seeds were produced in a HAuCl_4_ – Sodium citrate solution; different surfactant ratios yield different shapes	
2.5 CCNCB	1 : 25; +	—	*ca.* 920 nm	Gold nanorods, grown from CTAB-coated seeds in a growth solution containing CTAB : NaOL in 4.7 : 1 were used for this overgrowth protocol. Furter, Cu^2+^ ions were present in the growth solution; LSPR was estimated from spectrum	52

**3. CTAB : DDAB**					
3.1 NR	11–46 : 1; +	*W*: 10–24 nm	—	CTAB-seeds; “the dimensions were measured from SEM and TEM images by counting 200 particles.”	5
		*L*: 33–45 nm			
		AR: 1.9–3.3			
3.2 Dog-bone NR	23 : 1; +	*L*: 36–46 nm	—	CTAB-seeds	5
		*W*: 19–22 nm			
		AR: 1.6–2.4			
3.3 Dumbbell NR	23 : 1; +	*L*: 45–49 nm	—	CTAB-seeds	5
		*W*: 13–17 nm			
		AR: 2.9–3.4			
3.4 Elongated THH	8 : 1; +	*W*: 168 ± 15 nm	—	CTAB-seeds; shape yield >∼90%	5
		*L*: 198 ± 20 nm		Byproduct: bipyramids	
		AR: ∼1.2			
3.5 BP	7.7 : 1; +	Based on S(T)EM: *W*: 107 ± 45 nm, *L*: 382 ± 107 nm	619	CTAB-seeds	53
		Based on DLS: 144.9 ± 1.1 nm			
		AR: 3.8 ± 0.5			
3.6 BP	7.7 : 1; +	*W*: 266 ± 19 nm	593 (broad)	CTAB-seeds; the paper states a wrong CTAB amount in the experimental section (3.3 × 10^−6^ mole) and is corrected by the author after inquiry to 3.3 × 10^−3^ moles. The corrected surfactant ratio is listed here; broad LSPR peak can be an indication for a low shape yield of the bipyramidal nanostructures; sizes from TEM images, counting over 100 particles	54
		*L*: 644 ± 85 nm			
		AR: 2.4 ± 0.3			
3.7 BP	2–6 : 1; +	*W*: ∼382 nm	*ca.* 590 nm	CTAB-seeds; high shape yield (>∼80%, highest with low pH), pentatwinned and tetrahedral Au NPs obtained as reaction by-products	5
		*L*: ∼1.2 μm			
		AR: ∼3.1			
3.8 Spinous-like	1 : 1; −	—	—	No details were given for the formation of the CTAC coated seeds	50

**4. CTAB : DDAC**					
4.1 NR	6–23 : 1; +	*W*: 13–15 nm	—	CTAB-seeds; “Au NRs with low-yield products (spherical and cubic NPs)”	5
		*L*: 30–33 nm			
		AR: 2.0–2.5			

**5. CTAB : DDAI**					
5.1 NR	16–33 : 1; +	Only NR: *W*: 15–40	—	CTAB-seeds; faceted Au NR + reaction by-products (mostly spherical)	5
		*L*: 33–91			
		AR: 2.2			

**6. CTAB : sodium oleate**					
6.1 NR	0.96–7 : 1; +	AR: 2.3–3.5	*ca.* 700–900 nm	Seedless approach; increasing oleate leads to decreasing AR; “yields nearing 100%, negligible shape impurities”, shape yield ≥ 98%, standard deviation of diameter and length *ca.* 15%	55 and 56
6.2 Nanoribbon, nanobelt	1.9–12 : 1; −	*W*: 42 nm	—	CTAB-seeds; shape yield >90%, highest with lower CTAB : NaOL ratio	57
		Tickness (AFM): 23.9 nm, *L*: 1.6–40 μm			
		AR: large			
6.3 NR	3.7 : 1; +	*W*: 13–37 nm	*ca.* 700–850 nm	Seedless approach; “the shape yield approached 100%”	39
		*L*: 58–92 nm			
		AR: 2.5–4.5			
6.4 NR (‘cigarlike’)	4.7 : 1; +	*W*: 16 ± 1.3 nm	Near 950 nm	Overgrowth approach of nanorods as seeds; “TEM analysis reveals a very small percentage (less than 2%) of impurity particles”	58
		*L*: 80 ± 7 nm			
		AR: 5.0 ± 0.2			
6.5 NR (‘cigarlike’)	4.7 : 1; +	*W*: 20–61 nm	755–900 nm	Overgrowth approach of nanorods as seeds; “percentage of impurity particles (cubes, platelets, quasispheres, *etc.*) remains negligible in all samples”	58
		*L*: 90–146 nm			
		AR: 2.4–4.5			
				“Statistical analysis (up to 300 particles) was made to systematize the results of the TEM experiment”	
6.6 NR	6–3.8 : 1; +	*W*: 15–50 nm	650–1150 nm	CTAB-seeds; the calculation of the binary surfactant ratios used in this publication^59^ is based on the synthesis details provided in the supplementary information and not from the concentration ratios of the two surfactants stated in the text. To our understanding the concentration named in the text of 0.0126 M NaOL in the growth solution is calculated to be around 0.0078 M NaOL instead; “negligible shape impurities (less than 0.5% of the total number of nanoparticles)”; ‘sharp LSPR: fwhm = 110 nm at LSPR 857 nm’, very weak absorbance at 525 nm, which would point towards the presence of spheres	59
		*L*: 69–187 nm			
		AR: 3.8–7.6			
6.7 (truncated) elongated THH	4.86 : 1; +	*W*: 70–115 nm	<700 nm	CTAB-seeds; “negligible shape impurities (less than 0.5% of the total number of nanoparticles)”; ‘sharp LSPR: fwhm = 110 nm at LSPR 857 nm’, very weak absorbance at 525 nm, which would point towards the presence of spheres	59
		*L*: 114–174 nm			
		AR: 1.5–1.6			
6.8 Elongated THH	4.5 : 1; +	Lower aspect ratio than initial ETHH (AR *ca.* 3)	—	Used ETHH as reported by Ye *et al.*^59^ for overgrowth	60
6.9 Compass	4–11 : 1; −	*W* (central sphere): 55 nm	600–865 nm	Sodium citrate capped spheres were used as seeds; highest shape yield for compass shape at molar ratio 5 : 1; compass shape described as sphere with conical tips	61
		*W* (tip): 5 nm			
		*L*: 100 nm			
		AR: *ca.* 1.8			
6.10 NR	10–100 : 1; +	*W*: 9 ± 1 nm	750–1040 nm	CTAB coated seed; used dopamine as weak reducing agent; highest AR at CTAB : NaOL ratio of 20 : 1, synthesis of “monodisperse GNRs with high yield”	62
		*L*: 54 ± 7 nm			
		AR: 6.4 ± 1.2			
6.11 BP	20 : 1; +	*W*: 27–30 nm	750–950 nm	CTAC coated seed was used, along with hydroquinone as reducing agent; 90% shape yield	63
		*L*: 71–116 nm			
		AR: 2.7–3.8			
6.12 NR	1.25–40 : 1; +	AR: 2.3–4.1	644–1087 nm	CTAB coated seeds; different surfactant ratios result in different quality of nanorods. Also influenced by the amount of HCl added	64
6.13 BP	2000–500 : 1, +	*W*: 13–67 nm	531–1100 nm	CTAC coated seeds; aim to decrease CTAB concentration needed for synthesis; “yield of AuNBPs to 90% without purification”	65
		*L*: 18–270 nm			
		AR: 1.4–4.0			

**7. CTAB : sodium linoleate**					
7.1 NR	4.7 : 1; +	*W*: 19–82 nm	Shorter and longer wavelength than 700 nm	CTAB-seeds; this experiment was only conducted as supporting data, which leads to limited data availability; “high yield, albeit with slightly inferior size uniformity and shape purity [compared to NaOL]”	59
		*L*: 95–145 nm			
		AR: 1.7–5			

**8. CTAB : sodium stearate**					
8.1 NR, spheres	4.86–3.79 : 1; +	—	—	CTAB-seeds; shape yield: “very low”, “amount of spheroidal impurities is significant”	59

**9. CTAB : *n*-decanol**					
9.1 NR	3.4–3.7 : 1; +	*W*: 7.5 ± 1.5 nm	*ca.* 700 nm	CTAB-seeds; “colloidal AuNRs of small dimension were obtained in high yield, with small amounts of spherical impurities (<2%)”; “yield of 98%”; LSPR maximum was approximated from potted UV-vis spectrum	66
		*L*: 21 ± 3.5 nm; volume: (0.9 ± 0.2) × 10^3^ nm^3^			
		AR: 2.8			
9.2 NR	2.3–4 : 1; +	*W*: 17–25 nm	*ca.* 700–900 nm	CTAB-seeds; the dispersity was evaluated by two methods: optical fit of the LSPR (based on *ca.* 10^11^ particles) and through observation of a few hundred of particles on TEM images; factor *D* (“aspect ratio dispersity, defined as the standard deviation from the mean”): 8.5–16 (in general highest at highest *n*-decanol amount and lowest at surfactant ratio CTAB : *n*-decanol = 3.5); LSPR maximum was approximated from potted UV-vis spectrum	66
		*L*: 58–78 nm			
		AR from optical fit: 2.7–4.9; from TEM: 2.7–4.8			

**10. CTAB : oleic acid**					
10.1 Etched NR	52 : 1; +	*W*: 17 ± 6 nm	734 nm	CTAB coated seeds	10
		*L*: 44 ± 6 nm			
		AR: 2.9 ± 0.9			
10.2 Dog-bone NR	52 : 1; +	*W*: 18 ± 6 nm	516, 679 (strongest), 796	CTAB coated seeds; the paper states a wrong CTAB amount in the experimental section (3.3 × 10^–6^ mole) and is corrected by the author after inquiry to 3.3 × 10^–3^ moles. The corrected surfactant ratio is listed here; data from TEM images, counting over 100 particles	54
		*L*: 45 ± 8 nm			
		AR: 2.8 ± 0.7			
10.3 NR	52 : 1; +	Based on S(T)EM: *W*: 13 ± 2 nm, *L*: 42 ± 4 nm; based on DLS: 14.4 ± 0.7 nm (ref. 53)	522, 737	CTAB coated seeds	10 and 53
		AR: 3.4 ± 0.5 (ref. 53)			
		3.9 ± 1.2 (ref. 10)			
10.4 NM	35 : 1; +	Based on S(T)EM: *L*: 81–120 nm	680–744	A so-called double-seeding process was used for this synthesis. The surfactant ratio was the same for both growth solutions	10
		AR: 1.6			
10.5 NM	35 : 1; +	Based on S(T)EM: *W*: 83 ± 14 nm	749	CTAB coated seeds; double seeded process was used. Both growth solutions had the same surfactant ratio	53
		*L*: 118 ± 15 nm			
		Based on DLS: 97.4 ± 2.4 nm			
		AR: 1.5 ± 0.4			
10.6 Multiple twinned NR	3.9 : 1; −	*W*: 18–25 nm	Expected above 1100 nm	CTAB-seeds; the purity of the technical oleic acid used in these experiments was given as 90%. This was considered for the calculation of the surfactant ratio; at high aspect ratios the longitudinal LSPR peak was expected to appear close to the near infrared region for which the data was not obtained in the publication, expected at 1300–2800 nm; shape yield (based on TEM images): ∼90%	67
		*L*: 226–543 nm			
		AR: 10–25			
				Reduction yield (based on aqua regia digestion and ICP-MS analysis): ∼72%; more than 200 NR were counted for the size determination	
10.7 THH	3.5 : 1; +	Based on S(T)EM: *W*: 171 ± 20 nm, *L*: 233 ± 25 nm; based on DLS: 164.8 ± 5.6 nm	609	CTAB-seeds	53
		AR: 1.4 ± 0.2			
10.8 Elongated THH	3.5 : 1; +	*D*: 129 ± 27 nm	568 (broad)	CTAB-seeds; the paper states a wrong CTAB amount in the experimental section (3.3 × 10^–6^ mole) and is corrected by the author after inquiry to 3.3 × 10^–3^ moles. The corrected surfactant ratio is listed here; size data from TEM images, counting over 100 particles	54
		*L*: 180 ± 25 nm			
		AR: 1.4 ± 0.3			
10.9 Nanotriangle + nanoplates	1 : 7.6; −	—	—	CTAB-seeds; “Au nanotriangle and nanoplates were obtained in high yield”	67
10.10 NR	≈1 : 10; +	—	—	CTAB-seeds; “the STEM image shows some cubic and spherical impurities.”	54
10.11 Multiple twinned NR	1 : 15.2; −	—	—	CTAB-seeds; “decrease in the yield [of pentatwinned high-aspect ratio gold nanorods] (≈70%), uniformity, and size distribution” “yield of triangle and plate nanoparticles increased to ∼30%”	67
10.12 NM	1 : 19; +	*W*: 71 ± 12 nm	557, 760	CTAB-seeds; two-seeded synthesis approach; size data from TEM images, counting over 100 particles	54
		*L*: 108 ± 15 nm			
		AR: 1.6 ± 0.3			

**11. CTAB : oleylamine**					
11.1 NR	6.2 : 1; +	*W*: 15 nm	Larger wavelength than 700 nm	CTAB-seeds; “high yield, albeit with slightly inferior size uniformity and shape purity [compared to NaOL]”; this experiment was only conducted as supporting data, which leads to limited data availability	59
		*L*: 68 nm			
		AR: 4.5			

**12. CTAB : Pluronic L-64**					
12.1 NR	3.2 : 1; +	*W*: 18 ± 1 nm	518, 603, 695, 914 nm	CTAB-seeds; spherical impurities with higher Pluronic content	68
		*L*: 75 ± 7 nm			
		AR: 4.3			

**13. CTAB : Pluronic F-68**					
13.1 Concentric, rectangles	9.3 : 1; +	*W*: 26 ± 4 nm	513, 593, 682 nm	CTAB-seeds	68
		*L*: 53 ± 6 nm			
		AR: 2.1			

**14. CTAB : SDSn**					
14.1 Nanobelts, nanocombs	6.5 : 1; −	*W*: 40–200 nm	520–540 nm	Seedless approach	69
		*L*: several tens of micrometers			
		Thickness: 15–30 nm			
		Large AR			

**15. CTAC : BDAC**					
15.1 Earbuds	9–0.3:1; +	AR: 7–19	Beyond 1200	CTAB-seeds; “reasonable monodisperse”; AR determination *via* ImageJ	70
15.2 CVNCB	1 : 1.04; +	—	*ca.* 750 nm	Gold nanorods, grown from CTAB-coated seeds in a growth solution containing CTAB : NaOL in 4.7 : 1 were used for this overgrowth protocol. Furter, Cu^2+^ ions were present in the growth solution	52

**16. CTAC : (*R*)-BINAMINE**					
16.1 Chirally grooved nanoparticles	40 : 1; +	*W*: 73 nm–175 nm	*g*-factor maximum from 650–1300 nm	CTAB/*n*-decanol seeds; higher chirality with BINAMINE compared to BINOL as co-surfactant; spherical gold nanoparticles and nanorods were used for overgrowth as well as different aspect ratio rods and cuboids. Information on achiral-to-chiral transition	9 and 71
		*L*: 165–270 nm			
		AR: 1.5–2.3			

**17. C**_***n***_ **TAC : NaOL, *n*** = **10–18**					
17.1 NR	2.8–4.9 : 1; +	*W*: 12–66 nm	*ca.* 600–850 nm	CTAB-seeds; CTAC with chain length *n* = 16; the longer the alkyl chain, the higher the control over anisotropic growth; different chain lengths of CTAC were used; dimensions from at least 50 NR TEM images	6
		*L*: 42–106 nm			
		AR: 1.4–3.8			
17.2 NR	3.1–4.7 : 1; +	*W*: 21–66 nm	*ca.* 620–750 nm	CTAB-seeds; CTAC with chain length *n* = 18, also called TSAC; the longer the alkyl chain, the higher the control over anisotropic growth; dimensions from at least 50 NR (from TEM images)	6
		*L*: 59–135 nm			
		AR: 1.4–3.3			

a The presence of Au(i) in the growth solution is marked with a +, while the absence of a silver source is marked with a −.

b If no aspect ratio was given in the publication, it was calculated from the provided data for average length and width of the particles.

A plethora of shapes have been reported in the literature, sometimes with different names. The different morphologies of Au nanoparticles including their crystallographic details are summarized below ordered by the different shapes. Further, notable trends in the different synthesis approaches are mentioned.

**Fig. 2:**
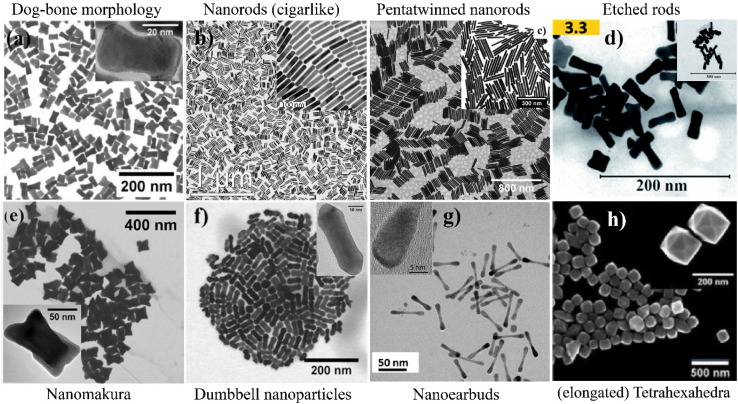
Overview of different elongated gold nanoparticle morphologies obtained from synthesis in binary surfactant mixtures: single-crystalline gold nanorods (NR, (b)), also described as cigarlike, are produced in a wide range of aspect ratios and quality in different binary surfactant mixtures, like here in CTAB : sodium oleate mixture;^59^ gold nanorods with a dumbbell morphology produced in a CTAB : DDAB mixture (f);^5^ etched nanorods grown in a CTAB : Oleic Acid (OA) mixture (d);^10^ nanorods with a dog-bone morphology from a CTAB : OA bound growth solution (a);^54^ gold nanoearbuds grown in a CTAC : BDAC binary surfactant mixture (g);^70^ (elongated) tetrahexahedra (THH) from a CTAB : DDAB mixture (h);^5^ pentatwinned gold NR grown in a CTAB : OA mixture (c);^67^ nanomakuras synthesized in a CTAB : OA growth solution (e).^54^

Single-crystalline nanorod synthesis can be facilitated with different binary surfactant mixtures.^5,10,72^ The first report of a growth solution using a binary surfactant mixture was published in 2003 by Nikoobakht and El-Sayed^4^ (see Table 2, 1.2) using CTAB and BDAC in a seed-mediated growth method, which yielded single crystalline gold nanorods with widths between 6–12 nm depending on the surfactant ratio. This work was expanded on for example in 2016 by Kim *et al.*^49^ (see Table 2, 1.4), where a surfactant ratio of 1 : 1 yielded the highest aspect ratio rods. Also, the synthesis with a binary surfactant mixture of CTAB and DDAB, yields Au nanorods. With increasing amount of CTAB in the binary surfactant mixture, Bandyopadhyay *et al.* yielded thinner and shorter NRs. Growth of these NPs were observed along the [001] direction, leading to {110} side facets and bound by {111} and {001} end facets^5^ (see Table 2, 3.1). The single-crystalline gold nanorods grown in a C_*n*_TAC : NaOL system by Ye *et al.*^6^ (see Table 2, 17.1) are enclosed predominantly by {310} planes on the end as well as the lateral facets. The first report of a seedless synthesis of gold nanorods in a binary surfactant growth solution came from Lai *et al.*^39^ (see Table 2, 6.3) and was further developed by Roach *et al.*^55,56^ (see Table 2, 6.1). In this one-pot reaction, the primary surfactant was CTAB and the co-surfactant sodium oleate (NaOL). Similarly, Ye *et al.*^59^ (see Table 2, 6.6) used NaOL to yield nanorods with diameters up to 70 nm in a “monodisperse” fashion employing a one-pot approach. This protocol was then further employed to study the effect of reaction temperature on the system while keeping the surfactant ratio of CTAB : NaOL constant.^72^ Khlebtsov *et al.*^58^ were able to reproduce the nanorods as reported by Ye *et al.*,^59^ and further performed overgrowth of these nanorods, which increased the dimensions (for both length and width) of “cigarlike” NR (see Fig. 2b and Table 2, 6.4, 5). The faceted surfaces of these particles are described to be enclosed by {100} and {111} planes at the rod ends, while {110} facets enclose the lateral, long surfaces. Wang *et al.*^73^ also used the gold nanorod synthesis proposed from Ye *et al.*^59^ for overgrowth towards nanoarrows in a single surfactant CTAC bound growth solution. Monodisperse gold nanorods were also grown in a CTAB : *n*-decanol surfactant mixture while small gold nanorods were used as seeds for further growth. In this method, symmetry breaking was individually optimized from consecutive further enlargement of the nanorods^66^ (see Table 2, 9.1, 2). This approach was taken further to achieve chiral gold nanoparticles by Gonzáley-Rubio and Mosquera *et al.*^9^ (see Table 2, 16). Overgrowth on these particles in a CTAC : (*R*)-BINAMINE binary surfactant mixture yielded particles with a complex surface morphology – wrinkles which were spiralling in a radial direction from the short axis with a tilt between 0 and 45°. These wrinkles were grown up to 20 nm in height from the nanoparticle seed surface and showed widths of 3–4 nm being separated by 2–3 nm.

One special form of nanorods is described as the dogbone morphology (Fig. 2a, see Table 2, 10.2). Bandyopadhyay *et al.*^5,54^ synthesized these using a Ag-assisted seed-mediated growth method with a binary surfactant system of CTAB and oleic acid (OA) in the molar ratio of 52 : 1. These nanoparticles were observed to be monocrystalline, having {110} side facets and {111} and {100} end facets. In the same publication^5^ a dumbbell morphology was reported (see Table 2, 3.3), grown in a CTAB : DDAB binary surfactant system (Fig. 2f). Here, the single crystalline particles have {110} side facets and {111} and {100} end facets. Later, Raghunathan *et al.*^10^ reported ‘etched rods’ (Fig. 2d, see Table 2, 10.1) also with a molar ratio of 52 : 1 CTAB to oleic acid. As presented in Fig. 2, these morphologies are rather similar and show the same facets.

Another shape that was grown with the binary surfactant mixture of CTAB and DDAB was elongated tetrahexahedra (ETHH, Fig. 2h, see Table 2, 3.4). These single-crystalline NPs were obtained at a surfactant ratio of CTAB : DDAB of 8 : 1 with a shape yield over 90%.^5^ Bandyopadhyay *et al.* were also able to synthesize this morphology using a surfactant mixture of CTAB : OA with a ratio of 3.5 : 1 (ref. 54) (see Table 2, 10.11). Further, Zhang *et al.*^60^ used ETHH as reported by Ye *et al.*^59^ for overgrowth in a 4.5 : 1 CTAB : NaOL surfactant mixture to yield ETHH with decreased aspect ratios (see Table 2, 6.8). Roy *et al.*^70^ showed the synthesis of single crystalline nanoearbuds (NEB, see Fig. 2g and Table 2, 15.1) with the binary surfactant system CTAC and BDAC showing three plasmonic peaks. When a single surfactant system was used, CTAC gave Au NR and spheres, whereas only BDAC yielded spheres. Only the combination of the two surfactants resulted in the formation of NEB. With varying surfactant ratios, the length and lateral dimension of the NEB could be adjusted. The highest aspect ratio of 15.3 ± 2.4 was obtained with a CTAC : DDAC ratio of 3 : 1. On the other hand, multiple twinned gold nanorods with a high aspect ratio (>10) were synthesized in a CTAB : OA, silver-free growth solution by Harper-Harris *et al.*^67^ (see Fig. 2c and Table 2, 10.11). The twin boundaries were observed using HRTEM and follow the major axis of the nanorods. SEAD pattern analysis in this work suggest that the nanorods are pentatwinned with five {100} side facets and five {111} facets on each end of the nanorods.

In 2018 Bandyopadhyay *et al.* reported a new nanostructure, namely: nanomakura,^54^ which have a similar morphology to dog-bone NR (see Fig. 2e and Table 2, 10.12). Nanomakura, however, are growing in all directions, which distinguishes them from these NR. These ‘nano pillows’ were synthesized through a Ag-assisted two-step seed-mediated approach with two growth solutions each containing a CTAB : OA surfactant ratio of 1 : 19.^54^ The reaction was further developed by Raghunathan *et al.*^10^ to yield these shapes with sizes from 80 to 120 nm with a constant aspect ratio of 1.6 in a CTAB : OA surfactant ratio of 35 : 1 (see Table 2, 10.4). Zhao *et al.*^69^ reported in 2008, the synthesis of nanobelts and nanocombs in a 6.5 : 1 CTAB : SDSn binary surfactant mixture (see Table 2, 14.1). The belts were grown along the 〈110〉 and 〈211〉 directions, depending on the reaction temperature. The combs consist of a 〈110〉 belt backbone with several 〈211〉 ‘teeth’ perpendicular to the ‘shaft’. In 2015, Xu *et al.*^57^ used a CTAB : NaOL mixture to yield nanobelts or nanoribbons (see Table 2, 6.2). The single crystalline ribbons grow preferentially along the 〈110〉 direction.

**Fig. 3:**
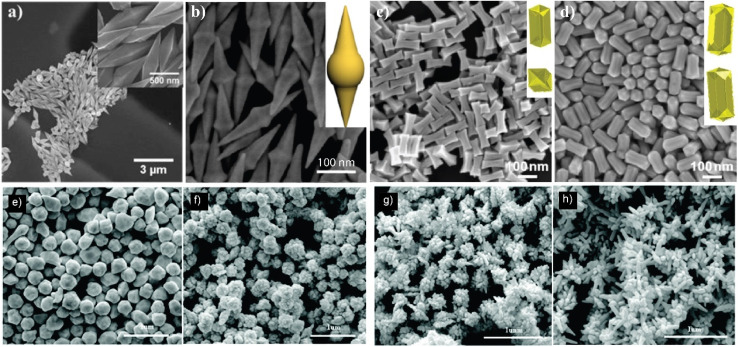
(a) SEM image of Au bipyramids synthesized with a CTAB/DDAB ratio of 2,^5^ (b) SEM image of nano-compass shaped gold nanoparticles produced in a CTAB : NaOL binary surfactant mixture and schematic illustration of the shape in the inset,^61^ convex nanocuboids (c) and concave nanocuboids (d) prepared through overgrowth in a CTAC : BDAC and CTAB : CTAC binary surfactant mixture growth solution, respectively,^52^ (e–h) SEM images of nanostructures with increased spikes when gradually increasing the CTAB : CTAC ratio.^50^

The synthesis of bipyramidal NPs with different sizes was achieved with a binary surfactant mixture of CTAB and DDAB^5,54^ (see Table 2, 3.6, 7). If the concentration of DDAB compared to CTAB was high (CTAB : DDAB ratio of 2 : 1), bipyramidal NP with a longitudinal length of 1.2 μm were obtained (see Fig. 3a).^5^ With a decreasing DDAB amount (CTAB : DDAB ratio of 6 : 1), the size of the bipyramids was roughly halved.^5^ With respect to the crystalline structure, the bipyramids exhibit (111) facets as well as 〈112〉 and 〈001〉 zone axes near the center, which are typical for decahedral or twinned nanoparticles with five-fold symmetry. These bipyramids are evidently face-centered cubic pentatwinned nanoparticles with sides bound by {111} and {100} facets and grown along the 〈110〉 direction.^5^ In a later publication of Bandyopadhyay *et al.*, the synthesis of bipyramidal NP was also reported with a CTAB : DDAB ratio of 7.7 : 1 (ref. 53 and 54) (see Table 2, 3.5, 6). Bipyramids were also obtained using a CTAC coated seed, and a growth solution using a 20 : 1 CTAB : NaOL mixture along with hydroquinone as reducing agent^63^ (see Table 2, 6.11).

Lee *et al.*^8^ used bipyramids as seeds to study the regrowth in three different binary surfactant mixtures: CTAB : BDAC, CTAB : CTAC and BDAC : CTAC. Of these, mostly the CTAB : CTAC showed overgrowth on the bipyramid tips (see Table 2, 2.1). Further, the tip morphology was different depending on the addition of AgNO_3_ and HCl to the growth solution. Regrowth to bipyramid-like shaped tips were of face-centered cubic and penta-twinned structure, showing (111) facets. Huang *et al.*^61^ reported compass shaped gold nanoparticles. These are described as spheres with two gradually thinning tips at the poles (see TEM image and inset in Fig. 3b, see Table 2, 6.9). Convex nanocuboids (CVNCB) were prepared by Zhang *et al.*^52^ in a CTAC : BDAC (ratio of 1 : 1.04, see Table 2, 15.2) overgrowth solution in the presence of Cu^2+^ ions. The CVNCB are single crystalline and show 24 well-defined convex high index {730} facets (see Fig. 3c). Also, concave nanocuboids (CCNCB, see Fig. 3d and Table 2, 2.5)^52^ were produced with an overgrowth protocol using a CTAB : CTAC binary surfactant mixture (ratio 1 : 25) in the presence of Cu^2+^ ions. The dihedral angle of 20.6° leads to the assignment of the 24 facets as {830}.

Zhang *et al.*^50^ yielded nanoparticles with different degree of spikiness depending on the surfactant ratio of CTAB : CTAC. At a ratio of CTAB : CTAC of 4 : 1, the resulting shape was described as quasi-spherical (Fig. 3e). When increasing the CTAB : CTAC ratio, first so-called popcorn-shaped gold nanoparticles form (Fig. 3f), (CTAB : CTAC ratio 1.5 : 1), rice paper pith shaped particles (Fig. 3g, CTAB : CTAC ratio 1 : 1.5) and at the lowest CTAB : CTAC ratio 1 : 4, spinous-like structures were observed (Fig. 3h, see Table 2, 2.3). The multiple pricks or tips of the spinous-like structures showed a lattice-fringe spacing of around *d*_111_ = 0.24 nm, which agrees with the (111) interplane distance of gold (fcc). Also, XRD patterns confirmed the high crystallinity of the nanoparticles. The size of the individual pricks was 110 nm in length and 60 nm in width. Similar structures were also formed in a CTAB : DDAB surfactant system.^50^

### 2.3 Anisotropic gold nanoparticles for biomedical applications

Depending on their morphology, the different anisotropic gold nanoparticles may be promising candidates for bio-medical applications. As this topic has been extensively reviewed elsewhere^40,74–79^ this section shall only focus on a few examples of bio-medical applications of anisotropic gold nanoparticles that were synthesized using binary surfactant mixtures.

Use of anisotropic gold nanoparticles for plasmonic refractometric sensing is practical, as the longitudinal plasmon band shows higher sensitivity compared to transverse plasmon resonances. Especially CCNCBs with a plasmonic refractometric sensitivity of 650 nm RIU^−1^ are promising candidates. Further, enhancement factors (EF) for SERS in the order of 10^6^ were reported for CVNCBs and CCNCBs.^52^ Gold nanoribbons demonstrate an enhancement factor in the same EF range.^57^ Nanobelts and nanocombs showed different Raman enhancement in SERS applications depending on the prominent surfaces.^69^ Spinous like-^50^ and compass^61^ shaped particles as well as gold nanoparticles with nanotips^51^ have also been used for SERS applications because their sharp tips work as ‘hot spots’ for localized near-field enhancement. Different gold nanoparticle geometries were covered with stimuli-responsive hydrogels as drug delivery vehicles, which was demonstrated with cytochrome C as model protein drug.^53^ Further, the gold nanoparticle shape was found to be an important parameter for cellular uptake as well as cell death.^54^ The use of gold nano bipyramids as theranostic agents was demonstrated with high contrast computed tomography combined with photothermal cancer therapy.^63^ To understand how to control the growth of such nanoparticles and tailor them for specific applications, it is important to understand their formation mechanisms.

## 3 Mechanistic discussion

Here, we summarize coherently the underlying mechanisms that drive the formation of anisotropic nanoparticles. In most of these experimental reports, the authors refer to mechanisms which have been hypothesized for nanoparticle growth in single surfactant solutions. Therefore, we present first a short overview of the mechanisms corresponding to the CTAB-containing seed-mediated synthesis and continue with insights from computer simulation studies. Then, we present the mechanisms for growth of anisotropic particles in the binary surfactant systems.

### 3.1 Mechanism of growth of nanoparticles in single surfactant systems – experiments

Seed-mediated growth of Au NPs in presence of CTAB is mechanistically complex to understand because chemical reaction, secondary nucleation and growth occur simultaneously. Moreover, these events are influenced by the additives, surfactant and synthesis conditions. One of the most established protocols for obtaining anisotropic structures is that of nanorods, hence Au NR synthesis is used to explain the mechanisms on an exemplary basis. In this context we draw the readers' attention to the previous reviews by Lohse *et al.*^1^ and Grzelczak *et al.*^2^ and Mosquera *et al.*^80^ From our viewpoint, the mechanism can be classified into three domains: (i) symmetry breaking that leads to anisotropy, (ii) evolution of the various shapes under the influence of reaction parameters and (iii) the evolution of shapes as a function of time.

For the evolution from a single crystalline ‘spherical’ seed to an anisotropic structure, the symmetry of the particle needs to be lowered through a ‘symmetry breaking’ event, which happens early in the synthesis.^41,81^ Further, the ratio of [HAuCl_4_] : [AgNO_3_] influences the seed size at which the symmetry breaking event occurs. For Au seed size of >4 nm, asymmetric “truncations” form, which are seen as the precursors for developing {110} facets upon further growth. Further, underpotential deposition (UPD) of silver on different facets can also facilitate symmetry breaking. UPD is characterized by deposition of 1–2 monolayers of silver metal onto gold metal at a lower potential than in the absence of gold. Since {110} facets have a higher UPD shift than {100} and {111}, the silver inhibits further growth especially there.^2,81^ Symmetry breaking for penta-twinned rods grown in a silver-free growth solution is explained based on a strain in the crystal lattice instead. By elongation of the penta-twinned seed in the unstrained direction, the particles grow as rods.^2^ The so-called ‘electric field model’ in a silver-free growth solution explains the anisotropic growth through the role of cationic CTA^+^. Adsorption of CTA^+^ as a double layer at the side facets results in a positive charge of the growing nanoparticle. The building units (Au^1+^ ions) are also associated within positively charged CTAB micelles and are hence transported to the growing tips, because of a lower surface potential over curved surfaces.^2^

In the “zipping” mechanism of a CTAB bilayer, the preferential adsorption of the surfactant onto lateral {100} edges compared to {111} faces is explained by different face stability as well as steric factors.^2^ For the growth of single crystalline, low aspect ratio gold nanorods, the role of silver is explained in three different mechanisms: firstly, UPD (see above) could ‘poison’ longitudinal surfaces and promote anisotropic growth towards rods. It is however challenging to explain the growth of other anisotropic shapes than nanorods with this phenomenon.^1,2^ Secondly, CTA–Br–Ag^+^ or AgBr_2_^−^ complexes could act as facet-specific capping agents. It is hard to distinguish between Ag^0^ and Ag^1+^ with (*in situ*) surface characterization methods,^1^ and differentiating between UPD and facet-specific capping agents is dependent on improved characterization techniques. The third mechanism refers to the role of CTAB micelles as a soft template for the gold nanorod formation. The micellar shape is altered from spherical to cylindrical through the seed as well as silver- and bromide ions. This could explain two consecutive growth processes: rapid anisotropic growth to a maximum aspect ratio (*ca.* 5–6), until ‘filling’ the soft template at first. Then, isotropic growth accompanied by lowering the aspect ratio^1^ follows. Tracking the growth of nanoparticles *in situ* would help understand the growth of gold nanoparticles. Small-angle X-ray scattering (SAXS) and X-ray absorption near edge structure (XANES) data showed that every nucleus would evolve into a nanorod. It may be noted that these methods provide the time-dependent growth of nanoparticle of the whole sample, rather than the specific shape evolution of a single particle. The combination of SAXS and time-resolved UV-vis spectroscopy showed that bromide ions (from CTAB as well as KBr) retard the depletion of Au^*n*+^. Cryo-TEM observations revealed a stochastic evolvement of seeds into rods. The individual seeds grow rapidly at different time points just like popcorn in the microwave.^1,82^

### 3.2 Mechanism of growth of nanoparticles in single surfactant systems – simulations

To support several of the experimental observations, this field has seen a host of modelling studies connected to the growth of nanoparticles in single surfactant systems. Meena and Sulpizi used molecular dynamics simulations to study gold slabs with three different facets – [100], [110] and [111] – in contact with a pre-formed CTAB bilayer and electrolyte solution. These authors observed that on all three gold surfaces, the initial CTAB bilayer was unstable and instead CTAB molecules formed distorted cylindrical vesicles spaced by water channels through which gold salt (AuCl^2−^) and Br^−^ ions diffuse toward the gold surface and facilitate the growth of gold nanorod.^83,84^ In another MD simulation study, the packing of CTAB molecules were found to be less dense on a curved gold surface compared to a flat gold surface.^85,86^ The loose packing of CTAB in the former case mimics that of the tip of growing gold nanorod, wherein the migration of [AuBr]^2−^ ions from solution to the gold surface is easier than along the flat surface. These findings suggest that surface curvature is an essential component of the anisotropic growth mechanism. Unlike the initial CTAB bilayer on gold surface, MD simulations show that sodium bis(2-ethylhexyl) sulfosuccinate (AOT) surfactant bilayer on gold surface remains stable on gold surface.^87^

MD simulation and sum frequency generation spectroscopy methods showed that butyl- and dodecyl-dimethyl benzyl ammonium bromide surfactants form ordered monolayer on gold surfaces.^88^ The role of polarizability of gold atoms on the adsorption of citrate molecules was investigated using Au [111] and [100] surfaces. A stable citrate crown formed around the nanoparticle with nonuniform surface distribution of citrate ions with the preference for Au(111) facets over Au(100) ones. Comparison of the results of citrate crown formation between polarizable and nonpolarizable gold models showed a difference in the citrate distribution on the surface of the gold nanoparticle.^89^ Polarization effects on the adsorption of CTAB on gold surface has been investigated using MD simulations.^90^

Dissipative particle dynamics (DPD) simulation was performed with coarse-grained model for CTAB with explicit attractive interaction between Au and Br^−^ ions to bind CTAB molecules onto gold surface *via* the electrostatic interaction between the cationic head group and adsorbed Br^−^ ions on the gold surface. The simulation showed that CTAB formed a compact bilayer structure with the inner layer having higher ligand density than the outer layer.^91^ In another MD simulation study with coarse-grained CTAB molecules, on [100], [111] and [110] gold surfaces, CTAB forms an irregularly connected bilayer structure where channels among bilayers provide direct ion access to the surface.^92^

Sambasivam *et al.* studied the self-assembly of cetyltrimethylammonium chloride (CTAC) on nanoparticles using coarse-grained approach.^93^ CTAC was coarse-grained using MARTINI force-field, and a 3.5 nm sized nanoparticle was constructed by assigning negative charges on 162 surface particles ranging from 0 to −2*e* chargers per surface particles. Compared to uncharged NP, the negatively charged NPs were covered with a bilayer structure because the positively charged CTA^+^ interacts with negatively charged NP surface forming the first surfactant layer. Tails of the first layer interacts with tails of free surfactant forming a second layer mimicking vesicle-like assembly with the NP in the core. In another study, a charge-neutral gold-nanorod with pre-assembled monolayer of CTAB in a coarse-grained description was investigated.^94^ In this model, the tail groups of CTAB were attached to gold surface while the CTA^+^ group was exposed to water giving a net positive charge to the NP-CTAB composite particle.

When CTAC is mixed with chiral co-surfactants, long worm-like micelles with chiral activity are formed. This has been demonstrated for CTAC : BINOL and CTAC : BINAMINE mixtures using MD simulations. When such chiral cylindrical micelles are used in the seed-mediated synthesis, the metal deposition follows the chirality of the micelles forming wrinkles on the gold nanorods. The average intergroove separation was comparable to the width of the chiral cylindrical micelles, suggesting that gold salt reduction and deposition occur at the available gold sites between surfactant micelles wrapping helically around a gold nanorod. This study demonstrated the MD-simulation inspired chirality transfer from surfactant micelles to the gold nanorod surface.^9^ These simulations give molecular level information on the capping and adsorption characteristics of single and mixed surfactant systems on gold facets.

**Fig. 4:**
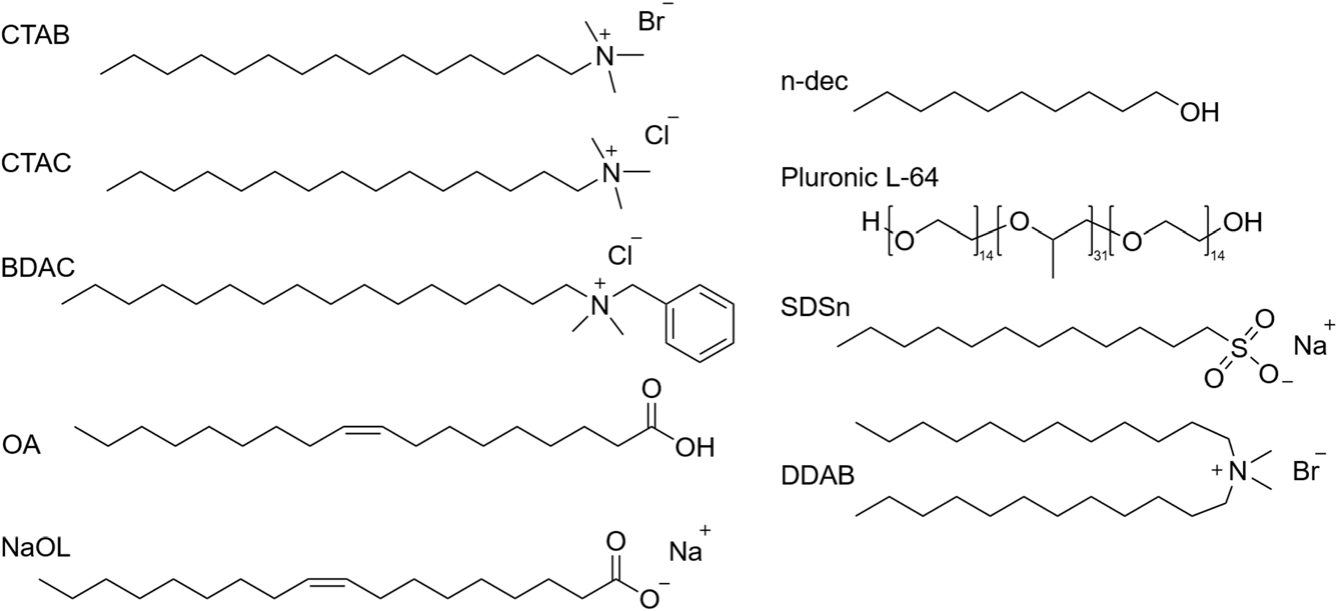
Skeletal formula representation of surfactants frequently used for the synthesis of gold nanoparticles: cetyltrimethylammonium bromide (CTAB), cetyltrimethylammonium chloride (CTAC), benzyldimethylhexadecylammonium chloride (BDAC), oleic acid (OA), sodium oleate (NaOL), *n*-decanol (*n*-dec), sodium docecylsulfonate (SDSn), didodecyldimethylammonium bromide (DDAB).

Although several studies have focused on attempting to understand the mechanisms behind the growth of anisotropic gold nanoparticles, many recent reports including the work by Bandyopadhyay *et al.*^5^ have shown enhanced size and shape tunability using binary surfactants. This necessitates summary of the various studies in this field and their approaches to reveal the mechanisms underlying growth. Our discussion here is classified based on the different binary surfactant mixtures employed in the literature and represented through their chemical structures in Fig. 4.

Herein, we classify the shapes obtained based on what kind of surfactant mixture is employed in the synthesis route – binary surfactants where surfactants do not act as reducing agent and binary surfactants where the co-surfactant also acts as an additional reducing agent along with ascorbic acid.

### 3.3 Binary surfactants (non-reducing)

#### 3.3.1 CTAB : DDAX

**Fig. 5:**
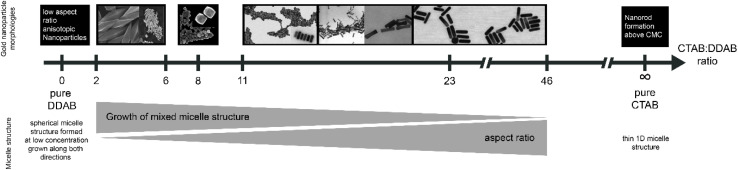
Overview of gold nanoparticle morphology resulting from synthesis at different CTAB : DDAB surfactant ratios as well as general trends for the micelle structures at different ratios, EM images from ref. 5.

In the binary surfactant system of CTAB and DDAB, a wide range of shapes could be synthesized with the seed-mediated growth process: concave cuboids, nanorods, dumbbell/etched/dogbone morphologies as well as elongated tetrahexahedra (ETHH) and bipyramids (see Table 2, 3, 5). The different morphologies seem to form preferentially in different surfactant ratio ranges as depicted in Fig. 5.

Pure DDAB (with a concentration of 17.3 mM) gave mainly ETHH with bipyramids as byproduct. CTAB : DDAB ratio in the range 2–6 yielded bipyramids, while a ratio of 8 gave ETHH. NR was the main reaction product for a ratio above 11 and the reaction produced shorter and thinner NRs with decreasing DDAB : CTAB ratio. When only CTAB was used as the surfactant, the shape was again dependent on the concentration of CTAB. As CTAB concentration is decreased, spheres were obtained instead of rods.^5^ These observations suggest that pure CTAB allows for the formation of NR when the concentration in the growth solution is above the critical micelle concentration of 8 × 10^−4^ mol L^−1^.^5,95^ These observations were reconciled by considering the changes in the shape of the mixed surfactant micelles.^5^

CTAB forms spherical micelles below a concentration of 100 mM. The addition of DDAB decreases the curvature in the CTAB : DDAB mixed micelle, changing the shape to rod-like.^5^ Neutron scattering studies showed that the longitudinal and transversal length of the micelle increased with decreasing CTAB : DDAB ratio.^96^ Further the packing density of trimethylammonium head groups on different facets was found as {110} > {100} > {111} through molecular dynamics simulations.^97^ Therefore, the {110} facet is more stabilized, slowing down the diffusion of Au atoms and growth. Au atoms are deposited on the facet {111} having lower surface passivation, leading to longitudinal growth. Though these experimental and simulation results suggest a possible mechanism of gold nanorod growth in CTAB : DDAB surfactant system, the mechanism of formation of other shapes listed above is unknown. One of the hypotheses suggests that at high DDAB concentrations, the mixed micelle structure becomes more convex, leading to formation of ETHH. Due to the high viscosity of the growth solution at high DDAB concentration, the growth was very slow, leading to a thermodynamically controlled ripening and growth of the Au bipyramids along the 〈110〉 axis.

Bandyopadhyay *et al.* have further investigated the effect of exchanging the bromide ion of the co-surfactant with chloride, yielding nanorods in a relatively low shape yield with spherical and cubic by-products. With increasing CTAB : DDAC ratio, the aspect ratio increased. With iodide as counter ion, the formation of gold nanoparticles was only observed at high CTAB : DDAI ratio, a too high DDAI amount rendered the reaction solution highly viscous and cloudy. The observed reaction results showed, that the shape tuneability of Au NP is greater with the secondary surfactant DDAB compared to DDAC and DDAI. These results suggest that adding DDAC and DDAI as co-surfactants destabilize the mixed micelle structure, possibly due to inefficient electrostatic screening. For gold nanoparticle growth this means with a less stable micelle, the surfactant molecules are less compact, which gives Au species easier access to the growing particle. In contrast, a stabilized micelle restricts the random growth as the Au species from the solution can not directly access the seed particle.^5^ Also, atomistic molecular dynamics simulations^97^ showed an important role of the Br-ion for the micelle adhesion on the Au surface. This explains the lower shape yield with reaction byproducts in the CTAB : DDAC and CTAB : DDAI system, compared to CTAB : DDAB.^5^ Further, the additional halide ions from the precursor, acids for pH adjustments or additional reactants do not seem to play an important role in the shape directing abilities for the synthesis of gold nanoparticles.^5^

#### 3.3.2 CTAC : BINAMINE

González-Rubio and Mosquera *et al.*^9^ used spherical gold nanoparticles and nanorods for the seeded growth of particles with a complex surface morphology (see Table 2, 16). These are described as intricate network of wrinkles, which are oriented in a radial direction with tilt angles between 0 and 45°. The special feature about these particles is their chiral optical activity, which arises from its handed wrinkle structure facilitated by a chiral co-surfactant. They found that the co-surfactant (*R*)-BINAMINE (*g*-factor: 0.2)††The *g*-factor is also called Kuhn's dissymmetry factor and is a unitless quantitative measure to compare different chiral structures. was directing the overgrowth towards stronger chirality than (*R*)-BINOL (*g*-factor: 0.002). The difference between the two surfactants is one hydroxy group that is exchanged by a primary amine moiety, which enables greater affinity with the other amine group present in the molecule.

The authors explain the growth based on the influence of the mixed micelle. Firstly, they template the growth of the chiral wrinkles grown around the seed particle and secondly, stabilize these features. This can be visualised by having mixed micelles coiled around the gold nanoparticles during growth. This hypothesis is also supported by the similar size range of the mixed micelle diameter (3.75 nm) and the wrinkle distances (2–3 nm). The micelles influence overgrowth by directing the diffusion of micellar aggregates which contain the building units for growth. This is however only observed when diffusion of the building units on the gold surface is slower than the rate of gold ion deposition. Further, the wrinkles were observed to grow the other way around when the (*S*)-enantiomer of BINAMINE was used for growth. Spheres as templates for overgrowth resulted in similar wrinkled structures, however with a lower chirality (*g*-factor: 0.003). González-Rubio *et al.*^9^ also studied the influence of the seed concentration. Further information on the development from achiral-to-chiral as well as using different aspect ratio nanorods for the chiral overgrowth was investigated by Zhuo *et al.* in 2022.^71^

#### 3.3.3 CTAB : SDSn

**Fig. 6:**
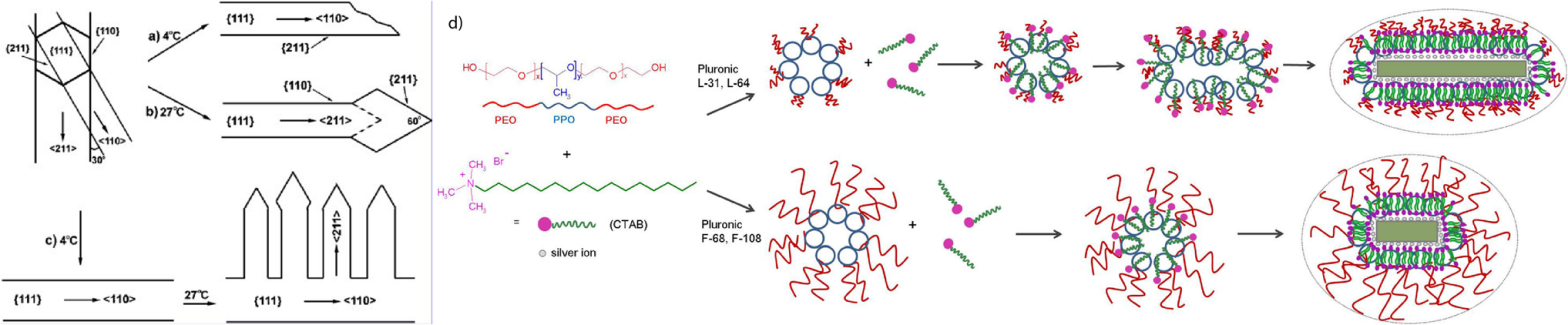
Proposed reaction mechanism for the formation of nanobelts at different reaction temperatures (a and b) and the stepwise reaction leading to anisotropic nanocombs (c);^69^ Schematic over the formation of different mixed micellar structures from CTAB and Pluronics (d). The length of the polyethylene oxide chains determine the mixed micellar shape and hence the resulting gold nanoparticle structure.^68^

Nanoribbons and nanocombs were synthesized in a CTAB : SDSn (sodium dodecylsulfonate) binary surfactant mixture (see Table 2, 14) by Zhao *et al.*^69^ It was observed that the growth direction was directly dependent on the reaction temperature. Zhao *et al.* proposed that the surfactant mixture plays a role as binary capping agents during the nanobelt growth. The cationic–anionic surfactant mixture can form a mixed surfactant film, which is adsorbed onto specific crystal facets. Here, the capping process would be influenced by the temperature and change the growth direction. This could result from different aggregation states in solution as well as the packing state of the mixed film and a proposed mechanism is illustrated in Fig. 6. The surfactants adsorb strongly onto the {111} planes at both temperatures. The adsorption strength on {211} facets is reduced at 4 °C, while at 27 °C the adsorption is less on {110} surfaces. Furthermore, the lateral nanobelts are only growing on one side of the ‘stem’ when the reaction temperature is increased, which leads to symmetry breaking.

#### 3.3.4 CTAB : Pluronics

CTAB together with different Pluronic triblock copolymers enable the synthesis of anisotropic gold nanoparticles, as demonstrated by Park and Song^68^ (see Table 2, 12, 13). The copolymers are constituted of polyethyleneoxide (PEO) –polypropylene oxide (PPO) – PEO blocks with varying hydrophilicities leading to their assembly into micelles, which are reported to form lamellar mesophases at high concentrations. When CTAB is present in addition, the Pluronics and CTAB form mixed micelles which are proposed to be either elongated (in case of short PEO blocks) or isotropic (with a large PEO corona), see Fig. 6d. These micelles then act as a soft template for the growth of gold nanoparticles.

It is proposed that during the growth of gold nanoparticles, a CTAB bilayer on the particle is gradually built up through the stabilized complex of Ag(i) and CTAB. Further, the growth is believed to proceed in the hydrophobic core of the Pluronics. The PEO subunits in the Pluronics enable reduction of Au(iii), while the PPO blocks adsorb onto the surface of the nanoparticles.

#### 3.3.5 CTAB : *n*-decanol

González-Rubio *et al.*^66^ showed that preparation of intermediate small gold nanorods (21 × 7.5 nm) in the reaction can lead to gold nanorods with increased control over size and aspect ratio. The authors point out that a seed which has already proceeded through symmetry breaking is a more reliable candidate to facilitate reproducible growth of anisotropic nanostructures. González-Rubio *et al.*^66^ uses *n*-decanol which results in worm-like or lamellar mixed micelles instead of spherical ones with CTAB only^98^ (see Table 2, 9). Further, the use of *n*-decanol as co-surfactant with CTAB leads to a decrease of the Krafft temperature of CTAB, which allows for synthesis temperatures as low as 16 °C.^66^

For the intermediate seeds, only a CTAB : *n*-decanol ratio of 3.4–3.7 : 1 yielded 98% gold nanorods and <2% spherical impurities. When less *n*-decanol was used, the shape yield was decreased. These intermediate Au NRs, or anisotropic seeds were further subjected to another growth solution containing CTAB : *n*-decanol in ratios between 1 : 2.3–4. The most monodisperse sample based on LSPR was obtained at a CTAB : *n*-decanol ratio of 3.5. It was further observed that the addition of *n-*decanol to the growth solution increased the monodispersity of the resulting gold nanorods. Decreasing the CTAB : *n*-decanol ratio results in a red shifted LSPR band, which became more intense. The authors further explored the influence of time, [HAuCl4] : [AgNO_3_] ratio as well as the [HCl] : [HAuCl_4_] ratio.

The co-surfactant *n*-decanol is assumed to change the passivation and stabilization behavior of CTAB aggregates on different facets of the gold seed, facilitating an increased efficiency in symmetry breaking. Here, CTAB is aggregating and adsorbing at high-index facets which prevents the supply for new building units and hence the growth on this surface. The co-surfactant *n*-decanol increases the aggregation behavior of CTAB when small gold nanorods are present. This mechanism was supported by diffusion-ordered NMR spectroscopy (DOSY) data, which showed that the self-diffusion coefficient of CTAB was lowered in the presence of small gold nanorods when *n*-decanol is present in the system. The reduced diffusion coefficient of CTAB is assumed to result from an increased fraction of CTAB that is adsorbed onto the gold nanorods in the presence of *n*-decanol. Further, it is assumed that *n*-decanol is also incorporated in the CTAB aggregates at the gold nanorod surfaces due to their matching self-diffusion coefficients. The authors further use these anisotropic seeds for growth and demonstrate that disconnecting symmetry breaking from growth leads to improved reproducibility and reliability of the synthesis.^66^

#### 3.3.6 CTAB : BDAC

In 2003, Nikoobakht and El-Sayed^4^ were among the first ones to synthesize gold nanoparticles using binary surfactant mixtures. They investigated surfactant ratios of CTAB : BDAC from 1 : 1.3 to 1 : 27 (see Table 2, 1). The reaction is described as a fast growth process (about 1 h reaction time) followed by a slow one up to 10 days, which was here called aging. The surfactant ratio influences the resulting plasmon maximum as well as the nanorod width. With increasing BDAC amount, the width of nanorods was roughly halved. Further, the reaction was adapted by gradual addition of the binary surfactant growth solution with a reduced gold precursor concentration. This led to continuous size enlargement in transverse and longitudinal directions. The authors also investigated the role of gold- and silver-ion concentration.

The authors proposed two different growth mechanisms: firstly, the surfactants can form a soft template, whose size is dependent on the surfactant concentration and the ionic strength of the growth solution. Here, the CTAB capped seed fuses with the surfactant micelle and growth is facilitated through diffusing gold atoms into the soft template. The other mechanism is based on new incoming gold atoms towards the lattice that are being protected by surfactant monomers from the solution. The authors describe the width enlargement (6–12 nm depending on the surfactant ratio) of the nanorods from the seeds (>4 nm). Hence, the growth is proceeding simultaneously in all directions. When a critical size is reached, the facets are large enough to enable significant surfactant binding. The final shape would then be determined by the growth rates on different facets in presence of surfactants. Tuning these is achieved by the supply of building units and surfactant concentration. It is assumed that CTAB is mainly found at the {110} side facets due to the larger binding affinity and BDAC would then be found at the rod ends. This is supported by the observation of the largest LSPR red-shift for the highest BDAC amount. The above-mentioned observations lead to the proposed mechanism of limited and slow growth in *x* and *y* direction of the nanorod (width) and faster growth in *z* direction (longitudinal, forming {110} facets). Here, the silver ions facilitate the binding of CTAB monomers on the longitudinal facets and therefore hinder growth along the width. As aging of the nanorods was only observed in the binary surfactant system, the CTAB : BDAC mixed micelle is attributed to create a more flexible template. Only spheres were obtained, when using pure BDAC in the growth solution. This points towards the necessity of CTAB in the growth solution to yield anisotropic particles, which can be attributed to the bromide counterion in CTAB, enabling a stronger bond with Ag.

**Fig. 7:**
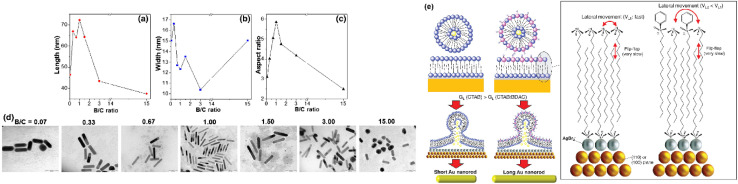
Influence of the surfactant ratio (here named *B*/*C* ratio; *B* for BDAC and *C* for CTAB) on the length (a), width (b) and aspect ratio (c) of the resulting gold nanorods (d);^49^ Schematic illustration of different micellar templates in a single surfactant CTAB system (e, left) and in a mixed micellar system from CTAB and BDAC (e, right) showing different packing densities. The different membrane fluidity results in a slower growth rate (*G*_L_) for the binary surfactant system. This is explained through the slower lateral movement in the mixed micellar system.^49^

Kim *et al.*^49^ studied the influence of the binary surfactant ratio on the resulting gold nanorods in a silver-assisted seed-mediated growth yielding gold nanorods with different sizes and aspect ratios (see Fig. 7). The surfactant ratio of 1 : 1 for CTAB : BDAC yields gold nanorods with the highest aspect ratio.

Further, Kim *et al.*^49^ concluded that CTAB is not essential for the growth of gold nanorods with a high aspect ratio if a bromide source is present in the binary surfactant mixture. Br^−^ was assigned to contribute to the protection of the side planes of the growing nanoparticles. Further, silver ions seemed to be essential in the growth solution for the symmetry breaking event and hence the development of anisotropic shapes.

To explain the shape directing properties of BDAC, the authors propose a growth mechanism based on altered membrane fluidity in the mixed micelles. BDAC has a larger headgroup compared to CTAB, which results in different packing densities in pure CTAB systems compared to when BDAC is present in addition (see Fig. 7e). Like cholesterol in phospholipid membranes, BDAC is believed to restrict the lateral movement of CTAB molecules and hence hinder the transport of the building units into the inner membrane by merging with the surfactant bilayer. Then the supply of building units to the lateral facets in the CTAB : BDAC system could be slower and hence promote anisotropic growth.

Further, the authors refer to the three proposed mechanisms for the growth of single crystalline gold nanorods in a silver-assisted seed-mediated growth process (see section above). Their observations, that increasing Br^−^ and Ag^+^ ion concentrations in the growth solution lead to blue-shifted LSPR peaks for the resulting nanoparticles does not agree with the mechanisms proposing selective facet protection through either Ag^0^ or a complex. There one would expect reaction products which show a red-shifted longitudinal LSPR peak, hence with a higher aspect ratio. Therefore, the authors propose the soft-template mechanism to guide the anisotropic growth. In detail, the silver and bromide ions would reduce the soft template size because of electrostatic interaction. Still, the authors observed that silver is essential for the evolution of the nanorods, so it was hypothesized that the specific facet protection of either Ag^0^ or a complex could be in effect during the symmetry breaking. Hence, all three proposed mechanisms could contribute to the development of the gold nanorods in the CTAB : BDAC binary surfactant system, just at different time-points.

**Fig. 8:**
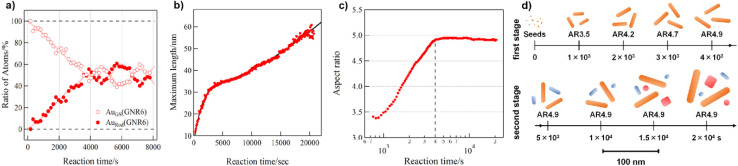
(a) Ratio of gold atoms in CTAB : BDAC binary surfactant based growth solution (Au_GS_) *vs.* gold atoms incorporated in the gold nanorods (Au_Rod_) over time from XANES data. (b) Calculated maximum length of the gold nanorods over time from fourier transformation of SAXS data. (c) Aspect ratio of the gold nanorods during the reaction progress derived from the position of the plasmonic peaks recorded with UV-vis spectroscopy.^48^ (d) Schematic growth mechanism of gold nanorods in a CTAB : BDAC binary surfactant system: in phase 1, all seeds grow longitudinally into nanorods with an aspect ratio of 4.9. In stage 2, only the nanorods with an ‘appropriate’ CTAB : BDAC surfactant ratio will continue growing longitudinal as well as transversal (orange), while other particles act as suppliers (blue) by ejecting Au building units. This can then lead to formation of shape impurities like spheres and cubes (red).^48^

In 2018, Hatakeyama *et al.*^48^ investigated the formation of gold nanorods *in situ* in a CTAB : BDAC system in a surfactant ratio of 1 : 2.5. When compared to a single surfactant system, the gold nanorods in the binary surfactant system take longer time to grow (2000 s *vs.* more than 20.000 s), which leads to the proposal of a different formation process. In essence, the gold nanoparticle formation is characterized by a building-in and releasing of gold building units, which makes the growth process slow. The authors combined time-resolved UV-vis, X-ray absorption fine structure (XAFS) and SAXS data to gain insights into the growth mechanism. The XANES data gives an insight into the distribution of gold atoms in the growth solution (Au_GS_) and bound into the growing nanoparticles (Au_Rod_) over time (see Fig. 8a). One can observe that more and more gold atoms are incorporated into the growing gold nanorods up until a reaction time of *ca.* 4000 s. After this timepoint the reaction seems to retard and the ratio of gold atoms in solution *vs.* in the crystal structure is fluctuating around 50%. As a reference, the ratio of gold atoms in the rods grown in a single surfactant CTAB system is completed after 2000 s at a ratio of over 90% of gold atoms in the rods. These rods however only yielded a lower aspect ratio. This leads to the conclusion that BDAC is facilitating the anisotropic growth as well as it slows down the reaction rate. Seen in the time resolved SAXS pattern, see Fig. 8b, the fast advancement of the maximum length stagnates after 4000 s, which agrees with the XANES measurements. After 4000 s, the maximum length still increases, however more gradual than in the beginning of the reaction and the aspect ratio remains the same (Fig. 8c). It must be noted here that the analysis method is biased to take particles with a large volume more into account and will contribute over proportionally to the increase of the maximum length.

The authors propose following mechanism in two stages: first, the nanorods grow anisotropically relatively fast towards an aspect ratio of *ca.* 4.9 with an average length of 33 nm (orange in Fig. 8d). Here the additional non-polar BDAC surfactant in the growth solution renders the mixed micelles to be smaller than for single surfactant CTAB micelles. In the second regime (after *ca.* 4000 s), the growth continues, however the aspect ratio remains. The nanorod population is divided in two groups: (1) continue growing isotropically and (2) being a gold supply by ejecting complex gold ions (*ca.* 60% of stage 1 NRs). The discrimination between the two population is based on the appropriate surfactant ratio between CTAB and BDAC at the individual gold nanorod. The generation of shape impurities, which means spheres or cubes (red in Fig. 8d), are assumed to develop from poorly coordinated seed- or supplier particles. It is further assumed that the release of the gold units from the supplier particles (blue in Fig. 8d) are happening anisotropically as well, which means from both ends as sort of a ‘backwards’ reaction. Further, the XANES data implicates that Au^+^ ions are not present in the growth solution, which suggests the presence of gold atoms or clusters which are coordinated by surfactants or other ions. Also, in the binary surfactant system all seed particles seem to grow into nanorods, which is not possible in a single surfactant CTAB system.

**Fig. 9:**
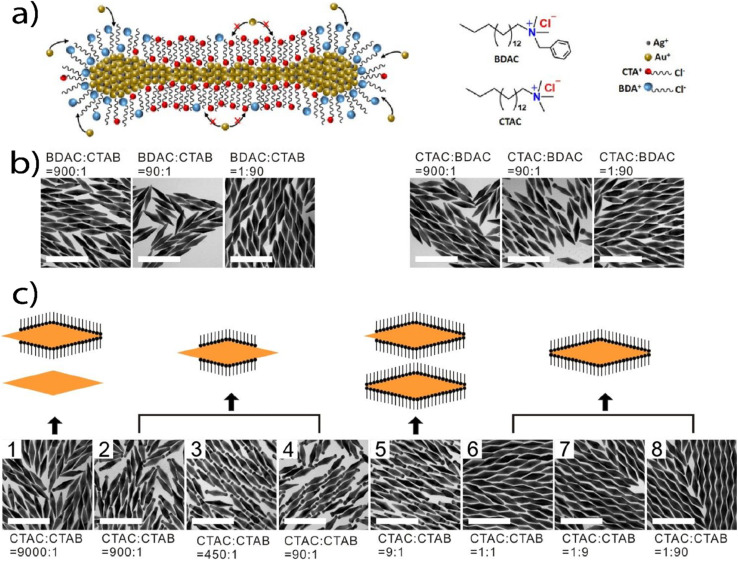
(a) Shape directing role of the binary surfactant system in the formation of nanoearbuds: preferential tighter packing of CTAC surfactant on the lateral sides compared to a more loose arrangement with BDAC at the ends.^70^ (b) Influence of the binary surfactant ratio on the resulting regrown shape of bipyramids: schematic representation of the binding of CTAB (black) and CTAC (exposed bipyramid area) in (c) which leads to different regrowth structures; scale bar is 200 nm.^8^

Using CTAC : BDAC system, Roy *et al.*^70^ synthesized nanoearbuds (NEBs). They proposed that the smaller headgroup of CTAC results in tighter packing of the surfactant molecules on the lateral facets of the seed, which hinders the transverse growth (see Fig. 9a). However, the BDAC headgroups are bulkier and can therefore not be tightly packed, leaving more space for the Au species to reach the growing particle. This assumption also explained the development of shorter and thicker Au NR with increasing BDAC concentration, as the amount of CTAC is too little to cover all lateral facets. Although the authors have mentioned the preferential arrangement of the different surfactants on the different facets, it is not trivial to understand why BDAC will not preferentially bind on the lateral surfaces. In regards to the counter ions of the employed surfactants in the formation of NEB,^70^ a chloride bound growth solution was used in this study. It must be pointed out that the Au seed however was synthesized with CTAB.

In another study, Zhang *et al.*^52^ developed an overgrowth protocol that produces concave and convex nanocuboids through different binary surfactant mixtures and Cu^2+^ ions. Further, nanocuboids with well-defined {100} facets were made when only CTAC was used as surfactant. When solely BDAC was employed, the overgrowth yielded irregular convex surfaces. CTAB overgrowth solution yielded faceted nanorods with truncated corners and concave surfaces. The tendencies of BDAC forming convex surfaces and CTAB making concave surfaces were cancelling each other out in the CTAB : BDAC binary surfactant mixture, which yielded irregularly shaped nanorods. Hence, it is assumed that when CTAC is combined with either CTAB or BDAC, well-defined facets can be formed (role of CTAC) which are either convex (through BDAC) or concave (through CTAB). In addition, the authors hypothesized with the help of XPS and *ζ*-potential measurements that the packing density of the surfactants is higher on high-index nanostructures like CVNCBs and CCNCBs compared to nanocuboids, which have mainly low index facets.

Spirin *et al.*^99^ compared the use of BDAC to BDTAC (hydrocarbon tail with *n* = 16 and *n* = 14, respectively) in a binary surfactant mixture with CTAB. The main difference lays within the shape yield of nanorods with BDAC, while the use of BDTAC enables the synthesis of longer nanorods, however with spherical impurities. The authors argue that the low yield of anisotropic nanoparticles in the BDTAC system is guided by the micellar template of the binary surfactant mixture.

Wadams *et al.*^100^ studied the effect of addition of a shape directing surfactant at different time points during the growth process. The co-surfactant BDAC was added into a CTAB growth solution to form a CTAB : BDAC ratio of 1 : 1.25, after 2–60 minutes of the seed addition. The longitudinal LSPR peak was blue-shifted the later into the growth process BDAC was added. The morphology change was observed in the TEM images: when BDAC was added in the beginning of the growth (0–5 minutes, = growth stages I and II, defined by Park^41^*et al.*) the gold nanorods are thin and long. Addition after 20 minutes results in short, truncated nanorods showing flat (100) end facets. Pure BDAC solution resulted in irregularly shaped nanoparticles which indicates that the initial formation of a CTAB–Au complex is essential for the formation of rods. Also 0.125 M CTAC was added to a 0.1 M CTAB growth solution at different timepoints of the reaction progress, tracking the role of the surfactant counterion. Also here, the addition of CTAC in the early stages of growth (0–5 minutes) had the largest impact. CTAC addition led to formation of cuboidal and spherical byproducts. The dimensions of the nanorods are comparable to the ones formed in pure CTAB solutions. The increase of CTAB concentration in the growth solution from 0.1 M to 0.225 M does not seem to be time-dependent and does not give significant changes in the LSPR response.

On basis of these observations as well as the proposed growth mechanism by Park *et al.*,^41^ the co-surfactant is proposed to mainly influence the micellar adsorption during the early growth stages where also the surface reconstruction happens. This influence is based on the observation that CTAB : BDAC mixed micelles are smaller compared to pure CTAB ones. According to Park *et al.*, the anisotropic growth in a single surfactant system is initiated when large enough surfaces are created for CTAB micelles to adsorb. This would be at a seed diameter of *ca.* 6 nm, which corresponds to the size of a CTAB micelle. With the smaller CTAB : BDAC mixed micelle, the restriction of transverse growth can be achieved earlier, leading to smaller width and longer length of the nanorods, resulting in a higher aspect ratio. When BDAC was added later (Stage III–V) in the growth process, the influence was smaller. Further, the influence of (100) facet passivation is visible in the formation of truncated tips of nanorods in the binary surfactant mixture.

The influence of the counterion is attributed to enhanced electrostatic screening of the micelles surface charge in case of CTAB compared to CTAC. This enables a higher adsorption density during the initial growth stages and promotes anisotropic growth. It is further argued that the chloride anion as counterion in BDAC and CTAC is not solely responsible for the morphology change, it comes from the whole surfactant molecule.

Lee *et al.*^8^ used purified monodisperse bipyramids as templates for overgrowth in different binary surfactant mixtures. As shown in Fig. 9, no overgrowth happens in a BDAC : CTAB mixture, while some was observed in CTAC : BDAC mixtures. However, in the CTAB : CTAC binary surfactant mixture, the ratio of the surfactants influences the regrowth, especially at the tips (Fig. 9c). This is explained by a greater binding affinity of CTAB compared to CTAC. When the CTAB : CTAC ratio is between 1 : 90 and 1 : 900 (Fig. 9c, Subfigure 2–4), an equilibrium between the surfactants is reached and the bipyramids are partly capped by CTAC. The distribution of the surface coverage is illustrated in Fig. 9c, where the black coverage of the surface represents CTAB, while the exposed surface represents CTAC coverage, which is assumed to be mostly at the tips. At this part of the particle, the binding preference difference between CTAB and CTAC is minimal, and the weaker protection leads to preferential growth. Further, the authors observed different shapes of the regrown bipyramid tips like spherical-, rod-, and bipyramid-like, influenced by HCl and AgNO_3_. Especially the regrowth to bipyramid-like tips in a growth solution with increased AgNO_3_ amount was only observed in the CTAB : CTAC binary surfactant mixture and not in the single surfactant system. The authors suggested that CTAC is more susceptible when it comes to the underpotential deposition of silver due to Cl^−^ as counter ion. The role of CTAB for this synthesis was later described by Sánchez-Iglesias *et al.*^101^ as the additive to the CTAC system for tailoring the aspect ratio at a fixed bipyramid volume.

#### 3.3.7 CTAB : CTAC

Song *et al.*^102^ have published a review article in 2023 on the use of CTAB : CTAC binary surfactant systems for the synthesis of high-index facet gold nanoparticles. The reader with interest toward electrocatalysis and SERS activity is directed to this review. Nanostructures with different spikiness were formed under different CTAB : CTAC ratios (see Table 2, 2). Spinous-like structures (see Fig. 3) were formed at a low CTAB : CTAC ratio (1 : 4). The morphological changes are accounted to the counter ion ratio of Cl^−^ and Br^−^ for the CTA^+^ molecule. The authors^50^ reference to the roles of the single surfactants as: CTAB aiding etching of highly curved tips and CTAC producing stellated features. CTAB has a higher coordination affinity onto gold than CTAC and the CTAB, which is assumed to adsorb onto the tips are then etching the sharp ends. With an increasing CTAC amount the etching is reduced, and more pricks are formed. In general, the trend was seen as growing longer and thinner bumps with decreasing CTAB : CTAC ratio.^50^

**Fig. 10:**
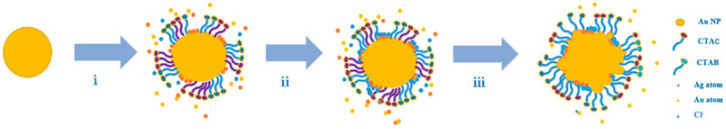
Schematic overview of the formation of nanostructures with tips from citrated coated gold nanoparticles.^51^

A similar approach was used by Mi *et al.*^51^ starting with 30 nm gold nanoparticles (citrate coated, named Au NP in Fig. 10) as seeds. CTAB : CTAC binary surfactant mixtures with ratios from 0.12–7 : 1 was used in this study. The growth of the tips is schematically represented in Fig. 10. The silver cations from addition of AgNO_3_ to the growth solution modifies the gold nanoparticle surface (step i). The influence from the different surfactants is based on the counter ion: namely chloride (from CTAC) or bromide (from CTAB). Cl^−^ ions can form a strong bond with Ag^+^ cations and will bind at sites which has silver ions present and will hence prevent gold atom migration and deposition at these sites (step ii). On the other sites, the gold nanotips are growing (step iii). The ratio of chloride determines the resulting nanoarchitecture.

### 3.4 Binary surfactants and reducing agents

#### 3.4.1 CTAB : NaOL

Ye *et al.* reported the use of co-surfactant sodium oleate (NaOL, see Table 2, 6) to overcome the difficulties of synthesizing gold nanorods with a uniform thickness in a CTAB mediated seeded growth method in 2013.^59^ The authors have grown ‘monodisperse’ gold nanorods with a diameter larger than 30 nm using this approach. It is argued that a too high CTAB concentration in seeded growth methods using CTAB alone, is preventing the formation of nanorods with a large diameter. When reducing to a CTAB concentration of 0.037 M in the growth solution in the presence of NaOL, rod diameters larger than 20 nm could be synthesized, while earlier established methods with CTAB concentrations of around 0.1 M mostly yielded diameters below 15 nm. When reducing the seed amount, they reported NR with diameters above 45 nm. Also, the effects of other parameters like pH in growth solution, the amount of seed particles and AgNO_3_ amount were investigated.^59^

NaOL is an anionic surfactant – its unsaturated structure is reported to act as a reducing agent to reduce Au^+3^ to Au^+1^ in the absence of the commonly used reducing agent ascorbic acid, shown through the discoloration of the gold salt. This hypothesis was further supported by replacing NaOL with sodium linoleate which possesses two unsaturated bonds, again leading to discoloration before adding ascorbic acid. Discoloration was not observed when sodium stearate was used instead, which does not have a double bond, thereby supporting the hypothesis. In addition, the shape yield of nanorods achieved through the CTAB : sodium stearate binary surfactant mixture is low and accompanied by many spheroidal impurities. The authors report that a low reaction pH (<1.7) is essential for producing uniform gold nanorods in the CTAB : NaOL system.

The authors argue that the low pH in the growth solution is necessary because NaOL is a basic salt. They however do not propose a mechanistic explanation to why additional hydronium ions or eventually the neutral form of the surfactant is vital for a uniform synthesis. Even though it was shown earlier that the NaOL will contribute to the reduction of Au^3+^, the mechanistic insights were limited. Raghunathan *et al.*,^10^ however investigated this aspect of the co-surfactant oleic acid, which is the acidic form of NaOL, more in depth (see sub-section below).

Furthermore, the authors claims to achieve higher tuneability and higher shape yield when reducing the CTAB concentration in the growth solution from the ‘standard 0.1 M’ to below 0.05 M in presence of the co-surfactant NaOL at acidic conditions.^59^ This statement is supposed to show the superior shape control of the CTAB : NaOL system, however it is biased in the sense that the optimized pH range for the binary surfactant system was used as argumentation basis. In addition, the amount of reducing agent ascorbic acid was adjusted in the binary surfactant system, to account for the earlier described role of NaOL as weak reducing agent. This decrease in amount of ascorbic acid will make it difficult to exclusively investigate the role of the co-surfactant, because the reaction kinetics will be affected as well. Increasing the amount of NaOL while maintaining the pH and all other growth conditions showed an increased diameter of the nanorods. This observation led to the suggestion that NaOL “might mediate the binding between CTAB surfactants and certain facets of growing NRs”.^59^

Liu *et al.*^72^ analysed the dataset from Ye *et al.*^59^ using a multivariate regression model, which takes the synthesis parameters CTAB (g), NaOL (g), seed (mL), AgNO_3_ (mL) and HCl (mL) as input and produces a model which predicts the aspect ratio (*L*/*D*) of the resulting nanorods. As one can observe from the resulting regression model equation, increasing amount of CTAB seems to result in nanoparticles with a higher AR, whereas a higher amount of sodium oleate leads to a smaller *L*/*D*. One must however mention that other parameters in this dataset, like the amount of seed, had a far larger influence on the resulting *L*/*D*. Further, the dataset from Ye *et al.*^59^ has only two datapoints for the different NaOL and CTAB concentrations. For a more conclusive model, additional CTAB and NaOL concentrations should be studied. Further, Liu *et al.* focused on the role of the reaction temperature while keeping the CTAB : NaOL ratio constant at 3.8 : 1. The main finding in this study was that both the length as well as the diameter of the nanorods increases with increasing temperature while the aspect ratio decreased. The authors argue along the lines that selective binding of CTAB as well as silver containing species on specific crystal facets direct the shape evolution. Hence Liu *et al.*^72^ are following the argumentation line suggested by Ye *et al.*^59^

**Fig. 11:**
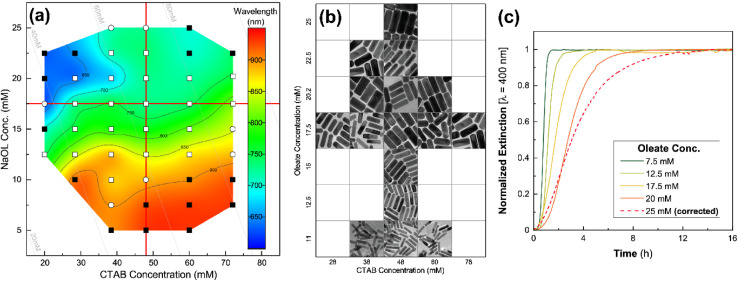
(a) LSPR wavelength of gold nanorods at different sodium oleate (NaOL) and CTAB concentrations in a one-pot seedless approach by Roach *et al.*^55^ The different points in the graph indicate the shape yield: empty square: shape yield >98%, empty circle: shape yield >90%, filled square: low shape yield. (b) TEM images of a 150 nm by 150 nm area of gold nanorods synthesized at different indicated NaOL and CTAB concentrations. (c) Kinetic data which reflects the influence of increasing NaOL concentration in the seedless synthesis in a CTAB : NaOL mixture; data at 25 mM [oleate] was corrected for turbidity.^56^

In another study, Roach *et al.*^55^ used a binary surfactant mixture of CTAB and NaOL in a seedless one-pot synthesis (the Au seeds are synthesized *in situ* in the growth solution by the rapid addition of sodium borohydride). They report that morphology control of the resulting gold nanorods happens *via* controlling the micellar soft template. Also here, it is discussed that sodium oleate possesses a double bond which can reduce Au^3+^ in solution to Au^1+^, visible through the clearing of the reaction solution.^55,59^ Roach *et al.*^55^ found that the aspect ratio changed more when varying the NaOL concentration compared to the CTAB concentration. This can also be seen in Fig. 11a, which shows a reduction of LSPR with increasing oleate concentration, indicating a decreasing aspect ratio. This decrease can be assigned to an increase in particle width, whereas the length was mostly unchanged as shown in Fig. 11b.

Fig. 11b also shows that the end-cap morphology changes form flattened, near-cylindrical tips to hemispheres at different surfactant ratios. The cap geometry is important due to its influence on the LSPR, which is red-shifted with decreased tip eccentricity.^103^

In a follow-up study by the same group,^56^ the reaction kinetics at different oleate concentrations were investigated. Two growth phases were proposed: initial rapid anisotropic growth and subsequent slower, more isotropic growth. The full width at half maximum (FWHM) of the LSPR peak decreased with proceeding reaction, which suggests increased monodispersity. As visible in Fig. 11c, the absorbance at 400 nm increases less rapid with increasing oleate concentration. This observation is referred to as a decreased Au^0^ evolution rate. The increasing negative charge of oleate is screening the positive charge of the quaternary ammonium headgroup. This leads to increasing packing density of the surfactants on the gold facets, which prevents the Au^+^ ions to reach the surface, hence slowing down the reaction.

From a mechanistic point of view, the two surfactants CTAB and NaOL have oppositely charged headgroups. As described in section on surfactants and micelle formation, mixed micelles with these conditions have a higher packing density in the micelle, because the head group charge is screened. In the particular case of CTAB–oleate, the micelles form rod-like micelles at lower concentrations, before branched structures are obtained.^104^ These observations were however not made in aqueous solutions but in ionic liquid bound systems. The authors are arguing that a rod-like micelle enables a high shape yield of gold nanorods. Further, they propose that the increased packing in the micelles through the binary surfactant system which reduces the flexibility in the micelle template can give access to larger gold nanorods. In this case, the radius of curvature is increased and rod-shaped nanoparticle synthesis is energetically favourable.^55^

**Fig. 12:**
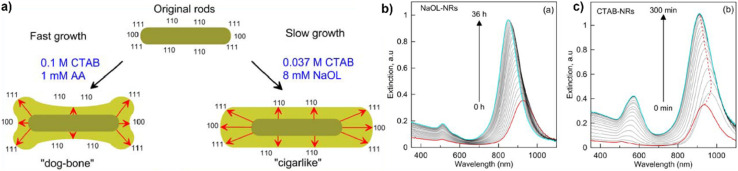
(a) Different overgrowth mechanism depending on the use of a CTAB based growth solution, which leads to overgrowth towards a dog-bone morphology and the binary surfactant mixture of CTAB and sodium oleate (NaOL) yielding cigarlike nanorods.^58^ Kinetic study of the overgrowth of gold nanorods with either a binary surfactant mixture of CTAB : sodium oleate (NaOL) with reduced ascorbic acid amount (b) or a single surfactant growth solution (c). The growth of the binary surfactant systems is slower compared to the single surfactant system and is characterized by a monotonic blueshift. The pure CTAB system also shows a red shift at first before the blue-shift.^58^

The binary surfactant system CTAB : NaOL can further be used in a protocol which uses the same growth solution twice, where the second growth phase is then called overgrowth. Here, one could start with the typical CTAB seed and produce ‘cigarlike’ nanorods with the first growth regime using the CTAB : NaOL binary surfactant mixture growth solution as discussed above by Ye *et al.*^59^ and Liu *et al.*^72^ Overgrowth in a binary surfactant mixture can ensure the “accurate cigarlike” shape of the formed nanoparticles, while having low level of impurities compared to overgrowth in a single surfactant system. The proposed mechanism during overgrowth is presented in Fig. 12a.

For explaining the effect of the binary surfactant mixture, the overgrown particles were compared with ones formed with a ‘traditional’ single surfactant 0.1 M CTAB growth solution, which grow into ‘dog-bone’ morphologies. Since the growth towards ‘dumbbell-like’ shapes shows a greater expansion on the {111} facets, it is assumed that these facets are more amenable to overgrowth compared to the lower index facets. Khlebtsov *et al.*^58^ mention the different reaction rates between the two approaches. In addition to stabilizing the NR crystal facets, NaOL is also reducing Au^3+^ ions. To balance this ‘extra reduction strength’, the amount of the weak reducing agent ascorbic acid (AA) was lowered in the growth solution, which decreases the reaction rate. This enables well controlled anisotropic growth as the reduction kinetics are lowered, as presented in Fig. 12b.

The overgrowth in the single surfactant CTAB growth solution is fast and does not follow a monotonic blue-shift of the LSPR (Fig. 12c). This fast anisotropic overgrowth yields mostly dumbbell-like nanorods associated with other shapes. The gold nanorods overgrown in the binary surfactant mixture however show a steady growth which is associated with the slow and continuous increase of extinction as well as a blue-shift of the LSPR (Fig. 12b). The LSPR plasmonic shift (Δ*λ*) in nm can be described with the following equation:
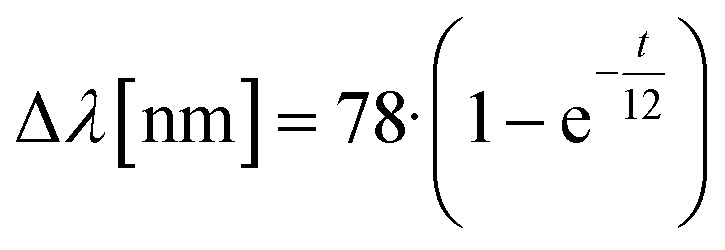
where *t* is given in [h]. This monotonic growth allows for the fine-tuning of the LSPR wavelength depending on the overgrowth reaction length down to a 1 nm shift, as every 20 minutes the LPSR shifts by 1 nm (within a window of 75 nm). Also, the plasmonic peak ratio between the longitudinal and transversal peak is larger for the binary surfactant system. The authors also comment that this precise control over the LSPR wavelength as well as reproducibility is not readily available when only changing the reaction protocol.^58^

In 2022 Wei *et al.*^64^ propose a growth model which is based on having seed particles of diverse size and crystalline structure. The different levels of NaOL concentration were observed to lead to different grown nanoparticle populations. At a low NaOL concentration, early symmetry breaking is followed by a popcorn like growth of few fast/growing particles. Moderate NaOL concentration results in independent monodisperse growth of the seed particles. At high NaOL concentrations the energy barrier is too high to have symmetry breaking in all seed particles and might happen at different timepoints. Hence, the growth leads to large interdependence of Au^+^ depletion and results in polydisperse particles.

#### 3.4.2 C_*n*_TAC : NaOL

Ye *et al.*^6^ use a bromide free surfactant mixture for the synthesis of gold nanorods (see Table 2, 17). This approach was the first to show that bromide bound surfactants are not essential for the tunable synthesis of gold nanorods. When the CTAC concentration is lowered, the gold nanorods grew larger in general. To tailor the nanoparticles further, the AgNO_3_ concentration, seed amount, and pH was varied.

**Fig. 13:**
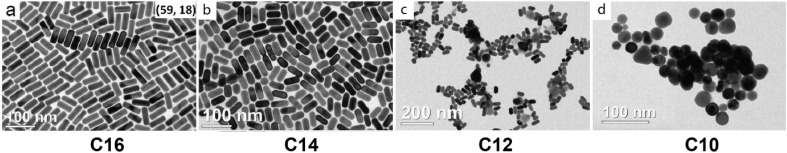
Gold nanoparticles yielded from synthesis of a CnTAC : NaOL mixture with decreasing chain length (as indicated in the figure), leading to lower control of anisotropic growth and monodispersity.^6^

The role of NaOL is once again assigned to its ability to reduce Au^3+^ to Au^1+^, which allows for more reproducibility. When lower purity (technical grade CTAC) was used, the nanorods were synthesized in comparable quality. The authors further demonstrated that even iodide concentrations up to 10 μM were tolerable. This means a two orders-of magnitude enhancement in tolerance against iodide impurities compared to conventional CTAB methods. The chain length of the aliphatic tail of the surfactant was changed from CTAC (C_16_) to TSAC (C_18_). Here, the lower limit for TSAC concentration along with NaOL is 0.03 M to yield nanorods. However, when the alkyl chain length was reduced (C_14–10_), the control over anisotropy and monodispersity disappeared (see Fig. 13).

The authors propose that these chloride rich growth solutions with alkyltrimethylammonium chloride have a synergistic effect with NaOL to enable anisotropic nanoparticle growth through facet stabilization. They further pointed out that instead of the oleate species, one can expect to see oleic acid molecules on the surface of the nanoparticles due to the low pH in the reaction solution.^6^

#### 3.4.3 CTAB : OA

Also the work by Raghunathan *et al.*^10^ highlights the new dimension for the role of surfactants. Oleic acid (OA) is used as co-surfactant with CTAB (see Table 2, 10), but its predominant role in this work is as reducing agent. Oleic acid has a double bond in the omega-9 position and can therefore be an electron donor, visible in the decolorization of the growth solution (Au^3+^ is reduced to Au^1+^) before adding the mild reducing agent ascorbic acid.^10,54^ The reduction strength through oleic acid was even increased by increasing the pH in the growth solution.^10^

With regards to the shape evolution, Raghunathan *et al.*^10^ aimed to connect growth to supersaturation generated due to reduction of Au^3+^ to Au^0^ in successive steps. In the study, the amount of weak reducing agent (ascorbic acid) was varied along with the pH, which alters the reduction potential. The reducing agent is necessary to reduce Au^3+^ to Au^1+^, which needs to be transported to the surface of the present seeds. At the surface, where also more reductants are present, Au^1+^ is then reduced to Au^0^. Consequently, there will be a build-up of Au^0^ supersaturation, which drives the surface integration of Au^0^ and the growth of the gold nanoparticles. Especially these last steps determine the shape of the particles. The reduction potential influences the kinetics of the reaction. With a high amount of reducing agent, the Au^0^ supersaturation built-up is high, and the monomer units integrate readily into the seeds. At this condition, integration of monomer units into the crystal is slower than the formation of Au^0^, which yields rough growth. On the other hand, at low reducing agent amount, the reaction to Au^0^ is slower and hence the buildup of supersaturation. Then, Au^0^ can integrate into the crystalline faces in an ordered way, which yields smooth shapes. The different observations in this study^10^ go hand-in-hand with the proposed mechanism: low supersaturations yield smooth nanorods. Increasing the supersaturation leads to rougher particles like etched rods. Increasing the supersaturation even further, shapes like nanomakura, spheres and multifaceted structures approach more the dendritic or spherulitic growth, as described in classical nucleation theory. This shape evolution of the different reaction conditions was elegantly shown in a ternary diagram which maps the resulting shape from different reaction conditions.^10^

At an earlier timepoint the observations by Bandyopadhyay *et al.*,^54^ led to a similar argumentation. Here, the growth solution turning form yellow to transparent led also to the assumption that OA is in fact acting as a weak reducing agent. In addition, the preferred growth onto the end facets of the nanorods was explained similar to the work from Khlebtsov *et al.*,^58^ which was giving mechanistic insight to the overgrowth of NR in a CTAB : NaOL system. Here, it was argued that the packing density of the CTAB : OA mixed micelle is higher at {110} and {100} facets compared to the {111} end facets. This yielded ‘dog-bone morphology’ nanorods. Further, when reducing the amount of the weak reducing agent ascorbic acid as well as oleic acid, it is assumed to decrease the reduction rate as well as diffusion rate of Au, which then leads to the formation of nanorods (with smooth surface).^54^

Special attention should also be directed to the facile spectroscopic method that was developed by Raghunathan *et al.* to track the growth of the gold nanoparticles and gives ideas about the different growth rates. The method revealed that the use of oleic acid in the growth solution alters the growth kinetics for different anisotropic shapes. Here again, the pH change in an oleic acid containing growth solution changed the growth rate constants by one order of magnitude. In the context of multivariate linear regression analysis of the presented dataset, the statistically relevant parameters were predicted. Here, oleic acid was the parameter with the highest influence on the position of the LSPR (red shift), as well as it decreased aspect ratio (AR) the most.^10^

In the work of Harper-Harris *et al.*^67^ the authors propose a growth mechanism which describes the growth from single crystalline CTAB-capped seeds (*ca.* 6 nm diameter) to pentatwinned gold nanorods. They argue that long-term stability of multiple twinned gold nanorods is achieved through the dense packing of the surfactant mixture of oleic acid and CTAB which is accounted to hinder the surface diffusion of gold atoms. The symmetry breaking event and formation of pentatwinned seeds is expected within the first few hours of the presence of seed particles in the growth solution. The evolution to form pentatwinned particles with {111} facets and {100} side facets is happening above a critical size. This is based on the observation that the growth solution is transparent and as well as the slow growth kinetics when OA is present in the growth solution. From the pentatwinned seed, the growth into the nanorod is assumed to be directed by the different affinity of surfactants bound to the facets. Since the surface energy of the {111} facets at the ‘tips’ is lower than on the {100} side facets, CTAB is expected to bind more strongly to the side facets and hence promoting their further evolution.^67^ The role of the co-surfactant oleic acid is focused on increased stability of the surfactant layer around the forming gold nanoparticles. A XPS study conducted by Harper-Harris *et al.*^67^ showed the presence of CTAB on the gold nanorod surface along with oleic acid. The presence of OA would then promote the stability of the CTAB bilayer.

The authors observed lower shape yield of gold nanorods when increasing the amount of oleic acid added to the growth solution and the viscosity increased. It was also proposed that the increased amount of OA is disrupting the CTAB bilayer micellar structure. This is then proposed to lead, also considering the increased viscosity, to a slower diffusion of gold atoms to the growing particle. Further, the reduction strength of the weak reducing agent can have an impact on the micellar structure. A high supersaturation of Au^0^ at the surface of the growing gold nanorods can lead to destabilization of the CTAB : OA micellar structure. This facilitates the deposition onto the side facets, yielding low aspect ratio gold nanorods. Alternatively, the negative charge of the carboxy group of oleic acid can lead to repulsion between the surfactant molecules.^67^

The synthesis of elongated tetrahexahedra with a CTAB : OA surfactant ratio of 3.5 : 1 (ref. 54), the change in resulting morphology is assumed to result from changes in the mixed micelle structure. At a relatively high amount of oleic acid (CTAB : OA ratio of ∼52 : 1) one obtains low aspect ratio nanorods with a dogbone morphology. At low OA concentrations, the mixed micelles are formed rod-like. Upon increasing the OA amount, the micellar structure is assumed to change into a convex shape, which aids the synthesis of the ETHH. This argumentation is based on an earlier work by the same group^5^ where an increasing concentration of the co-surfactant (in this case DDAB), enables the formation of convex micelles.^54^

**Fig. 14:**
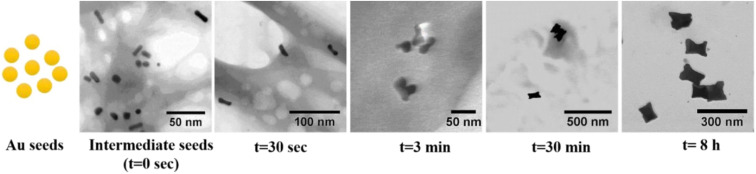
Time-resolved BF-STEM images which show the stepwise growth from intermediate seeds to nanomakura. Already after 3 minutes nanomakura in their final dimensions were observed, which suggests a ‘popcorn’ like growth mechanism.^54^

A two-seeded approach at a CTAB : OA ratio of 1 : 19,^54^ yields another new shape: nanomakura. These nanoparticles differ from the single seeded approach (yielding dog-bone morphology low aspect ratio nanorods), by having grown in all directions. The progression of the growth was observed at different time points, shown in Fig. 14. The intermediate seeds have grown into the longitudinal direction already. After 30 seconds the nanoparticles resemble a ‘bow-tie’ shape and some nanomakura in their final size were formed already after 3 minutes. The particles maintained the same shape and size both at 30 minutes and 8 h. These observations support the stochastic, sudden, ‘popcorn-like’ autocatalytic growth mechanism, which was proposed by Cortie *et al.*^82^

## 4 Challenges and opportunities

Various experimental recipes discussed in this article show that more than one additive is required for shape control of gold nanoparticles. From a thermodynamic point of view, shape anisotropy emerges because of surface free energy (*γ*) differences among the different facets of a freshly formed particle nucleus. For instance, an fcc unit cell with lattice constant *a* has *γ*[100] = 4, *γ*[110] = 4.24 and *γ*[111] = 3.36, in the units of *ε*/*a*^2^.^105^ Consequently, it is expected that the seed should take an octahedral or tetrahedral shape at equilibrium to minimize the {111} facets. However, both shapes have larger surface area per unit volume and therefore by Wulff's principle, a truncated octahedron shape is expected. Experiments have shown that alkane thiol coated gold nanocrystals formed in marks-type decahedral (Dh) and symmetrically twin-faulted truncated-octahedral (t-TO^+^) structures although theoretical predictions yielded, apart from these two forms, truncated-octahedral (TO^+^) form as well.^106^ This is due to the role of the surfactant (alkane thiol) in modulating surface free energies of the facets. When we extend this principle to binary and many surfactant systems, then depending upon the relative extent of adsorption of surfactants on different facets, many new shapes may be expected. All these arguments pertain to equilibrium conditions, yet the non-equilibrium factors bring in additional complexity in dictating the evolution of non-spherical shapes. For instance, in the nanoparticle growth solution, a range of time scales controls the overall shape evolution: reduction rate of metal ion species, transport rate of metal atoms to seed surface, transport rate of surfactants on seed surface, relaxation of surface atoms and so on. Complex interplay of these timescales resulting in numerous shapes as we change the experimental conditions (temperature, chemistry of surfactants, reducing agent, metal salt, *etc.*), is evident from experimental studies that have reported variety of different shapes. However, it may be emphasized that representations of these shapes as a function of reaction conditions in diagrams similar to phase diagrams will help enhance the visualization of the parameter space. Even though we understand the basic principles of nanoparticle formation (both thermodynamic and kinetic), we are not successful in predicting the shape *a priori* for a given experimental condition. This is because of lack of knowledge on the kinetic (rates) and thermodynamic parameters (solubility of metal, surface free energies, conformations of surfactants) which sometimes change during the synthesis.

Experimental sciences have advanced to a stage where certain events of particle formation such as growth are possible to observe and study, yet special resolution *in situ* in several other events (such as nucleation) is limited. Other studies using scattering techniques have yielded enormous but partial information on the formation of non-spherical shaped particles. In tandem, molecular dynamics simulation has been used to recreate experimental situation and study *in situ* growth of nanoparticle formation – mostly in the context of gold nanorods. These simulations can probe only nanosecond to microsecond timescales while experimental time scales are orders of magnitude higher. All these challenges call for a collaborative effort in documenting computational and experimental data in a common database. For example, surface energies of metal-surfactant systems have been widely studied in experiments and simulations. A database of these surface energy values will be very useful to explain or understand the qualitative role of surfactants on nanoparticle synthesis. Several experiments have also reported reduction rate of metal ions by weak reducing agents at different temperatures and solvent conditions. These kinetic data could be documented in the database as well. We also need new methods of doing molecular dynamics simulations that can bridge atom-level resolution to particle level resolution, and time scales reaching near experimental times. Scale-bridging and multi-scale approaches such as project EXAALT^107^ may be useful for such an endeavor. It may not be possible to build a generic MD package for all kinds of nanoparticles, but with so much scattered knowledge available on gold, it may be possible to build a multi-scale MD package specifically for gold nanoparticles by assembling datasets from density functional theory, all atom MD simulations and experimental studies. In this direction a dataset of codified gold nanoparticle synthesis protocols and particle size and shape information extracted directly from literature using natural language processing and text-mining techniques has been developed.^108^ As a user one may specify experimental conditions and the MD package should be able to deliver possible structures considering the equilibrium and kinetic limitations. Unknown parameters should be intrapolated using data science approaches such as machine learning. Therefore, plenty of opportunity exists for cross-disciplinary research activities encompassing synthesis, modelling, data analytics and wide range of applications in healthcare, energy and environmental applications.

## Data availability

No primary research results, software or code have been included and no new data were generated or analysed as part of this review.

## Author contributions

Katharina Zürbes: conceptualization, data curation, investigation, methodology, project administration, visualization, writing (original draft, review and editing); Ethayaraja Mani: data curation, funding acquisition, investigation, methodology, project administration, supervision, writing (original draft, review and editing); Sulalit Bandyopadhyay: conceptualization, funding acquisition, project administration, supervision, writing (original draft, review and editing).

## Conflicts of interest

There are no conflicts to declare.
